# Proteostasis and metabolic dysfunction characterize a subset of storage-induced senescent erythrocytes targeted for posttransfusion clearance

**DOI:** 10.1172/JCI183099

**Published:** 2025-03-11

**Authors:** Sandy Peltier, Mickaël Marin, Monika Dzieciatkowska, Michaël Dussiot, Micaela Kalani Roy, Johanna Bruce, Louise Leblanc, Youcef Hadjou, Sonia Georgeault, Aurélie Fricot, Camille Roussel, Daniel Stephenson, Madeleine Casimir, Abdoulaye Sissoko, François Paye, Safi Dokmak, Papa Alioune Ndour, Philippe Roingeard, Emilie-Fleur Gautier, Steven L. Spitalnik, Olivier Hermine, Pierre A. Buffet, Angelo D’Alessandro, Pascal Amireault

**Affiliations:** 1 Université Paris Cité, INSERM, BIGR, Paris, France.; 2 Université Paris Cité, Institut Imagine, Laboratory of Cellular and Molecular Mechanisms of Hematological Disorders and Therapeutic Implications, INSERM, Paris, France.; 3 Department of Biochemistry and Molecular Genetics, University of Colorado Denver, Anschutz Medical Campus, Aurora, Colorado, USA.; 4 Proteom’IC facility, Université Paris Cité, CNRS, INSERM, Institut Cochin, Paris, France.; 5 Plateforme des Microscopies, Infrastructures de Recherche en Biologie Santé et Agronomie, Programme Pluriformation Analyse des Systèmes Biologiques, Tours, France.; 6 Laboratoire d’hématologie générale, Hôpital Universitaire Necker Enfants Malades, Assistance Publique des Hôpitaux de Paris (AP-HP), Paris, France.; 7 Université Libre de Bruxelles, Hôpital Erasme, Département d’Hématologie, Brussels, Belgium.; 8 Department of Digestive Surgery, Hôpital Saint-Antoine, AP-HP, Paris, France.; 9 Department of Hepatobiliary Surgery and Liver Transplantation, Hôpital Beaujon, AP-HP, Clichy, France.; 10 U1259, Morphogenèse et Antigénicité du VIH et des Virus des Hépatites, INSERM, Université de Tours and Centre Hospitalier Régional Universitaire de Tours, Tours, France.; 11 Department of Pathology and Cell Biology, Columbia University, New York, New York, USA.; 12 Département d’Hématologie, Hôpital Universitaire Necker Enfants Malades, AP-HP, Paris, France.; 13 Service des maladies infectieuses et tropicales, Hôpital Universitaire Necker Enfants Malades, AP-HP, Paris, France.

**Keywords:** Cell biology, Hematology, Cellular senescence, Proteomics, Ubiquitin-proteosome system

## Abstract

Although refrigerated storage slows the metabolism of volunteer donor RBCs, which is essential in transfusion medicine, cellular aging still occurs throughout this in vitro process. Storage-induced microerythrocytes (SMEs) are morphologically altered senescent RBCs that accumulate during storage and are cleared from circulation following transfusion. However, the molecular and cellular alterations that trigger clearance of this RBC subset remain to be identified. Using a staining protocol that sorts long-stored SMEs (i.e., CFSE^hi^) and morphologically normal RBCs (CFSE^lo^), these in vitro aged cells were characterized. Metabolomics analysis identified depletion of energy, lipid-repair, and antioxidant metabolites in CFSE^hi^ RBCs. By redox proteomics, irreversible protein oxidation primarily affected CFSE^hi^ RBCs. By proteomics, 96 proteins, mostly in the proteostasis family, had relocated to CFSE^hi^ RBC membranes. CFSE^hi^ RBCs exhibited decreased proteasome activity and deformability; increased phosphatidylserine exposure, osmotic fragility, and endothelial cell adherence; and were cleared from the circulation during human spleen perfusion ex vivo. Conversely, molecular, cellular, and circulatory properties of long-stored CFSE^lo^ RBCs resembled those of short-stored RBCs. CFSE^hi^ RBCs are morphologically and metabolically altered, have irreversibly oxidized and membrane-relocated proteins, and exhibit decreased proteasome activity. In vitro aging during storage selectively alters metabolism and proteostasis in these storage-induced senescent RBCs targeted for clearance.

## Abstract 2

*In vitro* aging during cold storage selectively alters metabolism and proteostasis in Storage-induced MicroErythrocytes, targeting this distinct subset of senescent cells for clearance after transfusion.

## Graphical abstract

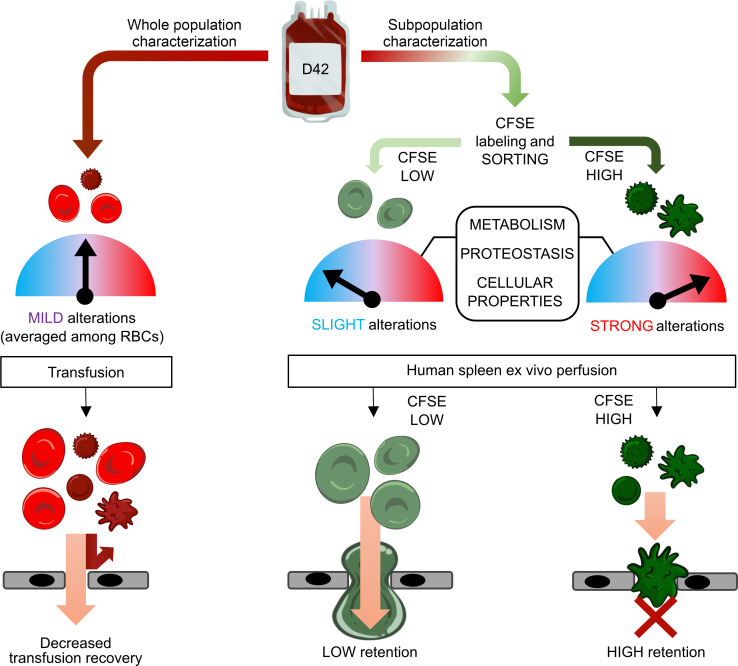

## Introduction

The worldwide annual collection of approximately 119 million blood units is necessary to treat the large number of patients requiring transfusion. In most European countries, whole-blood donations are processed into RBC concentrates and stored at 4°C in saline-adenine-glucose-mannitol (SAGM) solution for up to 42 days. Despite slowing of their metabolism during refrigerated storage, RBCs continue to age in vitro. This time-related decline shares similarities with physiological senescence in vivo (1). One important difference between these two aging processes is that storage-induced senescent RBCs accumulate alterations in the confined and protective environment of the storage bag, thereby (temporarily) avoiding clearance from the circulation; in contrast, newly senescent RBCs in vivo are continuously cleared from circulation. Multiple storage-related RBCs modifications have been described, reflecting the deterioration in their quality (2, 3). As examples, energy metabolism progressively diminishes (4–7), with decreased intracellular levels of ATP (8–10) and antioxidants (11), followed by increasing oxidant stress (3, 12). Thus, refrigerator-stored RBCs are less able to cope with oxidative stresses generated during storage, leading to increased lipid and protein oxidation (1, 6, 13–17). These metabolic and oxidative stresses progressively modify multiple RBC properties, including increased surface exposure of phosphatidylserine (PS) and endothelial cell adherence, decreased osmotic resistance and deformability, and altered morphology (18–21). The evolution of storage lesion markers in individual RBC units during aging in vitro varies greatly, with donor sex, age, and genetics influencing end-of-storage oxidative and spontaneous hemolysis in vitro and posttransfusion RBC recovery and hemoglobin (Hb) increments in vivo (22–25).

Although recent prospective studies did not show a survival advantage when transfusing RBC concentrates stored for less than 10 days (vs. standard of practice) (26–30), the storage lesion remains a matter of concern, as it is responsible for the rapid clearance of a substantial proportion of transfused RBCs, thereby decreasing transfusion efficacy (25, 31–35). Nonetheless, these studies suggest that only a subset of the stored RBCs is sufficiently altered in vitro to be recognized and then cleared, after transfusion in vivo, by the recipient’s mononuclear phagocyte system.

Decreased intracellular ATP (36–39), one component of the RBC storage lesion, negatively correlates with transfusion recovery, indicating that in vitro markers can inform on posttransfusion outcomes. However, this metabolite was measured at the whole-population level in RBC units; thus, its evolution in individual RBCs is unknown. Similarly, storage-induced morphological alterations are a cellular marker of posttransfusion clearance. Indeed, storage-induced microerythrocytes (SMEs; comprising type III echinocytes, spheroechinocytes, and spherocytes) accumulate during storage, vary between donors, negatively correlate with transfusion recovery in healthy volunteers, and are preferentially cleared from the circulation in vivo in a mouse transfusion model (40). However, the molecular and cellular abnormalities occurring in this distinct subset of morphologically altered RBCs, triggering their posttransfusion clearance, remain to be identified. Using a simple carboxyfluorescein diacetate succinimidyl ester (CFDA-SE) staining protocol (41), combined with flow cytometric sorting of RBCs stored for 35–42 days (referred to below as long-stored RBCs), preparations were obtained containing either more than 90% SMEs (i.e., carboxyfluorescein succinimidyl ester^hi^ [CFSE^hi^]) or more than 95% morphologically normal cells (i.e., CFSE^lo^), thereby allowing in-depth comparisons of their properties.

The current study characterized SMEs and compared them with morphologically normal long-stored RBCs and morphologically normal RBCs stored for 3–12 days (referred to below as short-stored RBCs). To this end, molecular phenotypes were identified using omics approaches (i.e., metabolomics, redox-proteomics, proteomics), and cellular characteristics were analyzed using multiple assays (i.e., deformability using a spleen-mimetic filter, adhesion properties, PS exposure, osmotic resistance, intracellular ATP concentration, proteasome activity). Finally, their circulatory capabilities were evaluated using an ex vivo human spleen perfusion model.

## Results

### CFSE^hi^ RBCs are SMEs that can be sorted by flow cytometry.

**Figure 1:**
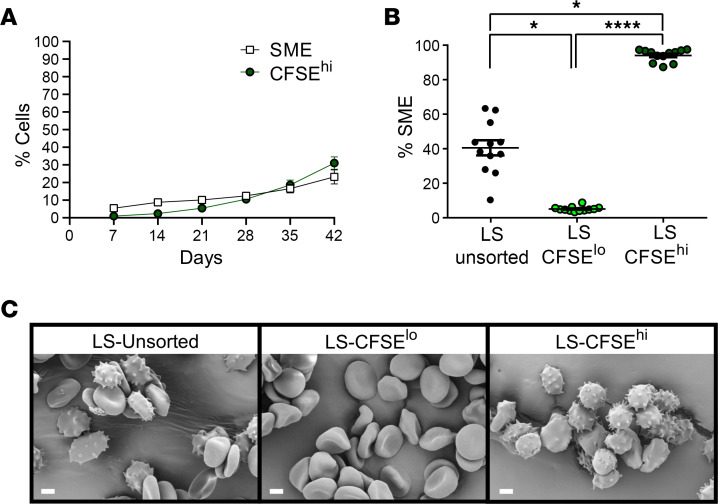
CFSE^hi^ RBCs are storage-induced micro-erythrocytes that can be sorted by flow cytometry. (**A**) Weekly quantification of storage-induced microerythrocytes (SMEs) (white squares) and CFSE^hi^ RBCs (green circles) in RBC concentrates stored in SAGM solution at 4°C for 42 days (mean ± SEM of 8 RBC concentrates). (**B**) Proportion of SMEs in CFSE-stained, long-stored, unsorted (LS unsorted) and flow-sorted CFSE^lo^ (LS CFSE^lo^) and CFSE^hi^ (LS CFSE^hi^) RBC subsets. Data are represented as individual points with mean ± SEM of 12 RBC concentrates. (**C**) Representative scanning electron microscopy images showing typical RBC morphology of CFSE-stained RBCs. Scale bar: 2 μm. In **B**, *P < 0.05, ****P < 0.0001 by a Friedman’s 1-way ANOVA followed by Dunn’s multiple comparison test (*n* = 12).

CFSE^hi^ RBCs were quantified weekly throughout storage in 8 RBC concentrates collected from healthy human donors and stored in SAGM solution. Consistent with our previous data (41), mean CFSE^hi^ RBC (minimum to maximum values ± SEM) accumulation during storage ranged from 1.0% (0.3%–2.8% ± 0.3%) on day 7 to 31.1% (11.6%–45.2% ± 3.5%) on day 42, with marked interdonor variability (Figure 1A). The kinetics and amplitude of CFSE^hi^ RBC accumulation were similar to the SME subset previously quantified by imaging flow cytometry. Quantifying SMEs in the sorted CFSE-stained long-stored RBC subsets (35–42 days of storage) showed that the CFSE^lo^ subset contained 4.9% SMEs, whereas the CFSE^hi^ subset contained 93.7% SMEs (Figure 1B). Scanning electron microscopy confirmed that sorted CFSE^lo^ and CFSE^hi^ subsets were highly enriched in morphologically normal RBCs (discocytes, echinocytes I and II) and SMEs (echinocytes III, spheroechinocytes, spherocytes), respectively (Figure 1C). These data confirm that highly enriched preparations of prone-to-be-cleared SMEs and also of long-stored morphologically normal in vitro–aged RBCs can be obtained, allowing comparison of their properties.

### Metabolomics identifies subset-specific metabolic alterations in long-stored CFSE^hi^ RBCs during aging in vitro.

**Figure 2:**
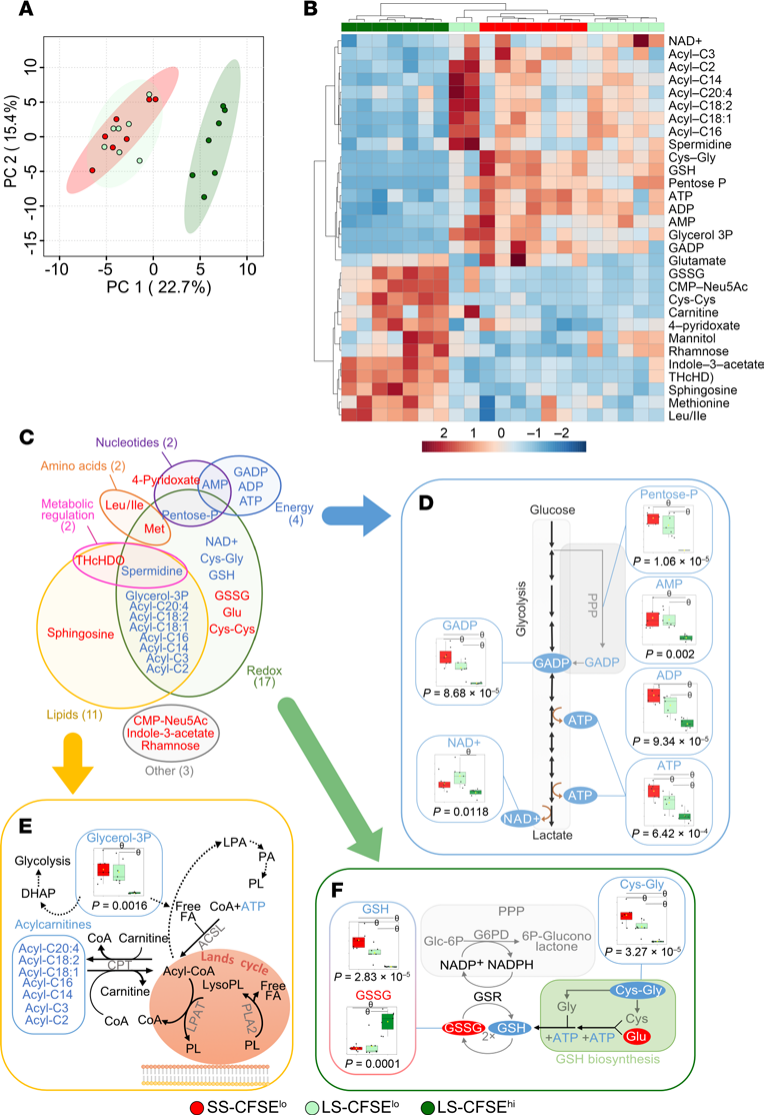
Metabolomics identifies subset-specific alterations in the metabolism of long-stored CFSE^hi^ RBCs during aging in vitro. (**A**) Principal component analysis (PCA) of metabolomics data on flow-sorted short-stored CFSE^lo^ (SS-CFSE^lo^, in red), long-stored CFSE^lo^ (LS-CFSE^lo^, in light green), and long-stored CFSE^hi^ (LS-CFSE^hi^, in dark green) RBCs. (**B**) Hierarchical clustering analysis of the 30 metabolites whose levels vary among the 3 groups by 1-way ANOVA followed by Tukey’s multiple comparison test. (**C**) Schematic distribution of the 28 metabolites whose levels vary significantly when comparing long-stored CFSE^lo^ and CFSE^hi^ RBC subsets. (**D**–**F**) Overview of glycolysis (**D**), lipid repair (**E**), and glutathione (**F**) pathways, highlighting key metabolites whose levels vary among the 3 groups. In **C**–**F**, the metabolites that show significant increases (red font) and decreases (blue font) in long-stored CFSE^hi^ RBCs (vs. long-stored CFSE^lo^ RBCs) are shown. Arrows represent a single metabolic step in the pathways, and dotted lines represent multiple steps. *P* values of 1-way ANOVA followed by a FDR correction are indicated under each graph, and θ represents a significant difference found by a positive Tukey’s post hoc test between groups. Abbreviation definitions are detailed in Supplemental data (Supplemental Methods, *Metabolomics and redox-proteomics*).

Metabolomics analysis compared short-stored CFSE^lo^ (stored for 3–12 days) and long-stored CFSE^lo^ and CFSE^hi^ subsets (stored for 35–42 days). Principal component analysis (PCA) illustrated the clear separation of the CFSE^hi^ subset from the 2 CFSE^lo^ subsets (Figure 2A). Heatmap representation of the 30 identified metabolites differing significantly between the 3 subsets also showed this separation, because individual CFSE^hi^ samples clustered together, whereas short-stored and long-stored CFSE^lo^ RBCs were much more similar to each other (Figure 2B). Indeed, when comparing short-stored and long-stored CFSE^lo^ subsets, only 8 metabolites differed significantly, including small variations in energy and redox metabolites for long-stored RBCs (Supplemental Figure 1; supplemental material available online with this article; https://doi.org/10.1172/JCI183099DS1).

However, when comparing long-stored CFSE^lo^ and CFSE^hi^ subsets, 28 metabolites differed significantly, with major differences in redox, lipid, nucleotide, amino acid, energy, and metabolic regulation (Figure 2C). For energy metabolism (Figure 2D), significant decreases in steady-state metabolite levels in the glycolytic and pentose phosphate pathways were observed in long-stored CFSE^hi^ RBCs, including hexose-phosphate (isomers), GAPDH (GADP), NAD^+^, and pentose phosphate (isomers), accompanied by decreases in the total adenylate pool, comprising high energy ATP, ADP, and AMP. Regarding lipid metabolism, glycerol-3-phosphate (glycerol-3P; implicated in energy and lipid processes) was significantly decreased, along with 7 different acyl-carnitines that participate in lipid recycling through the Lands cycle (Figure 2E and Supplemental Figure 2). For redox metabolism, oxidized metabolites significantly increased, including glutathione disulfide, cysteinylcysteine (Cys-Cys; an oxidized form of cysteine), and glutamate (Glu), accompanied by very low levels of NAD^+^, reduced glutathione (GSH), and its precursor, cysteinylglycine (Cys-Gly; Figure 2, C and F). These data suggest that the decreased level of various metabolites, such as ATP, GSH, and carnitines, which were previously identified at the whole-population level, are also distributed unevenly among individual RBCs and mainly affect the SME subset.

### Redox proteomics identifies a subset-specific capability to resist storage-induced oxidative stress during aging in vitro.

**Figure 3:**
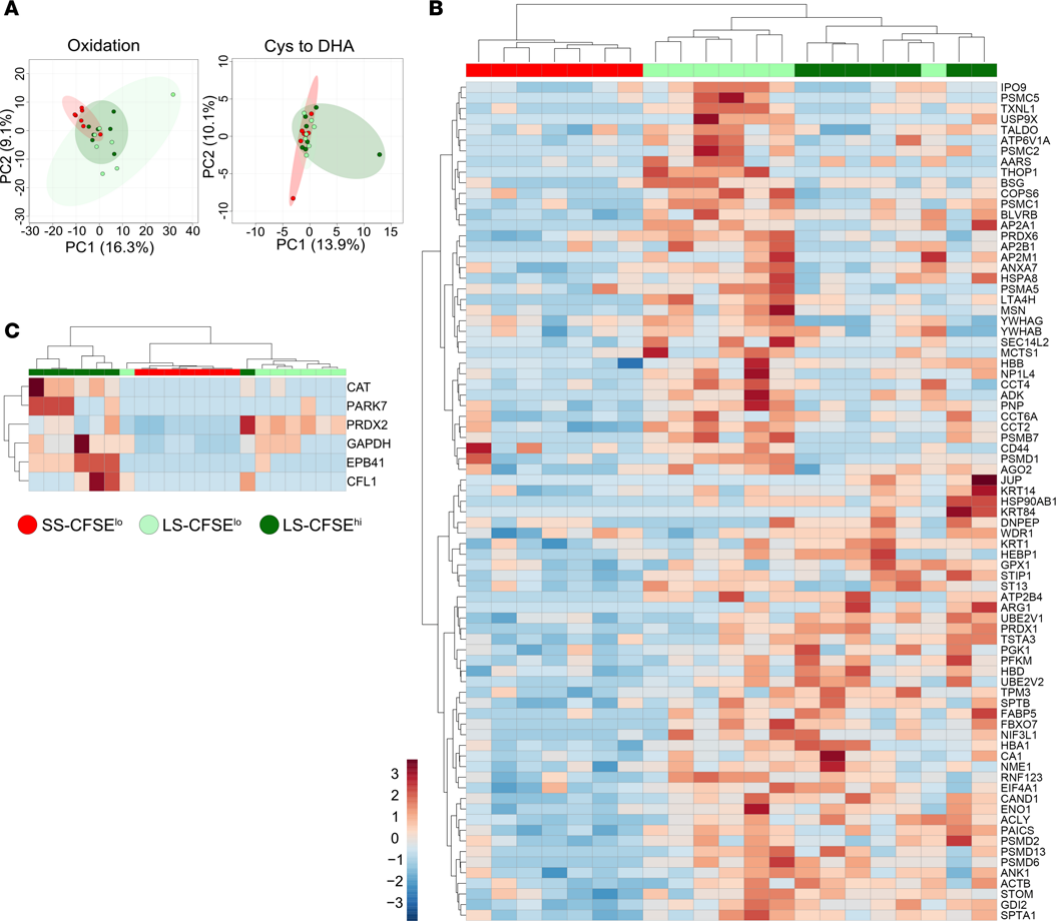
Redox proteomics identifies a subset-specific capacity to resist storage-induced oxidative stress during aging in vitro. (**A**) PCA of reversible (“oxidation”) and irreversible (“Cys to DHA”) protein oxidation data obtained from flow-sorted short-stored CFSE^lo^ (SS-CFSE^lo^, in red), long-stored CFSE^lo^ (LS-CFSE^lo^, light green), and long-stored CFSE^hi^ (LS-CFSE^hi^, in dark green) RBCs. (**B**) Hierarchical clustering analysis of the 79 proteins (of a total of 659 proteins analyzed, representing 11.7%) with significant reversible oxidation across the 3 groups by 1-way ANOVA followed by Tukey’s multiple comparison test. (**C**) Hierarchical clustering analysis of the 6 proteins (of a total of 176 proteins analyzed, representing 3.6%) with significant irreversible oxidation across the 3 groups by 1-way ANOVA followed by Tukey’s multiple comparison test.

Posttranslational redox modifications were quantified for reversible oxidation (especially of cysteine and methionine) and irreversible oxidation (e.g., cysteine converted to dehydroalanine via β-elimination of thiols) in short-stored CFSE^lo^ and long-stored CFSE^lo^ and CFSE^hi^ subsets. Unsupervised PCA illustrates the effect of storage on reversible oxidations in both long-stored CFSE^lo^ and CFSE^hi^ subsets, as compared with short-stored CFSE^lo^ RBCs (Figure 3A, left). However, no global differences were observed for irreversible oxidations (Figure 3A, right). Significant reversible oxidations were detected for 77 proteins, whereas 6 proteins were irreversibly oxidized.

Heatmap representation of the 77 proteins with reversible oxidations confirmed the impact of storage but also revealed subset-specific oxidized proteins when comparing long-stored CFSE^lo^ and CFSE^hi^ RBCs (Figure 3B). Reversibly oxidized proteins (Supplemental Table 1) derive from 6 main families: proteostasis (29%), cytoskeleton (11%), Hb (3.6%), antioxidant system (8%), transport (6%), and glycolysis (6%).

By heatmap representation of the 6 irreversibly oxidized proteins, most CFSE^hi^ samples clustered together, separately from the short-stored and long-stored CFSE^lo^ subsets, which clustered together (Figure 3C). Increased irreversible oxidation of 4 proteins was detected only in the CFSE^hi^ subset (CAT, PARK7, CFL1, EBP41), while 2 proteins (PRDX2, GAPDH) were irreversibly oxidized in both long-stored CFSE^hi^ and CFSE^lo^ subsets (Supplemental Table 1). A second, independent proteomic analysis comparing membrane and cytosol preparations from long-stored CFSE^hi^ and long-stored CFSE^lo^ RBCs was conducted to confirm the identity of the irreversibly oxidized proteins. Heatmap representation of the top 10 proteins with irreversible oxidations showed that most CFSE^hi^ samples clustered together in both preparations, but significant irreversible oxidation was detected only for 1 protein (ADK) in the membrane fraction of the CFSE^hi^ subset (Supplemental Figure 3). Thus, even if these 2 independent experiments did not identify irreversible oxidation of the exact same proteins, they did identify that irreversible protein oxidation mainly affected CFSE^hi^ RBCs, supporting the concept that SMEs have a reduced capability to handle and repair storage-induced oxidative stress.

### Proteomics identifies a subset-specific membrane relocation of proteins in long-stored CFSE^hi^ RBCs, notably impacting proteins of the proteostasis family.

**Figure 4:**
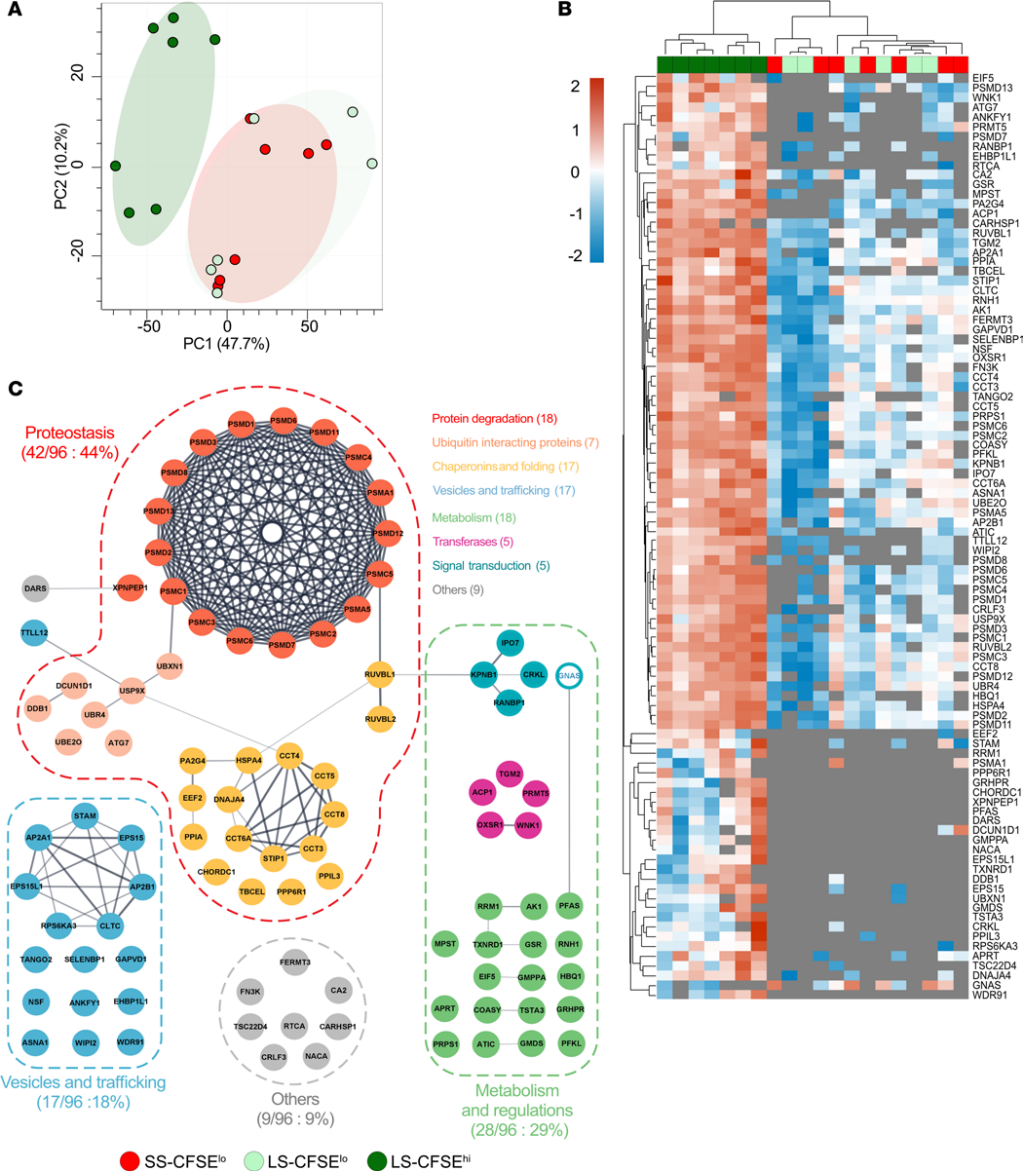
Proteomics identifies subset-specific membrane relocation of specific proteins in long-stored CFSE^hi^ RBCs, notably affecting proteins in the proteostasis family. (**A**) PCA of proteomics data obtained from membrane preparations isolated from flow-sorted short-stored CFSE^lo^ (SS-CFSE^lo^, in red), long-stored CFSE^lo^ (LS-CFSE^lo^, light green), and long-stored CFSE^hi^ (LS-CFSE^hi^, dark green) RBCs. (**B**) Hierarchical clustering analysis of the 96 proteins that significantly differ between the 3 groups following Pearson’s clusterization and a FDR correction without data imputation (proteins detected in at least 70% of samples were considered). A *z*-score scale calculated from copy number/cell shows each protein level, while lack of detection is represented by a gray area. (**C**) Interaction network analysis for proteins increased (full filled circle) and decreased (white, transparent, inner circle) in membrane preparations of long-stored CFSE^hi^ RBCs, as compared with long-stored CFSE^lo^ RBCs. Gray lines represent physical interactions between proteins. This network was realized using Cytoscape StringApp 3.9. Colored dotted lines represent the main protein families identified (relative proportion within the 96 significant proteins), and colored circles represent functional groups within a family.

Proteomics analysis compared short-stored CFSE^lo^ RBCs with long-stored CFSE^lo^ and CFSE^hi^ subsets. Proteomics of whole, intact RBCs revealed no significant differences among these 3 groups (Supplemental Figure 4). However, proteomics of the isolated RBC plasma membranes (i.e., “ghosts”) identified major differences between these 3 groups. By PCA, the long-stored CFSE^hi^ subset was clearly separated from the CFSE^lo^ subsets (Figure 4A). Heatmap clustering confirmed the uniqueness of CFSE^hi^ RBCs (Figure 4B), revealing expression differences of 96 proteins between the long-stored RBC subsets. However, no significant differences were seen when comparing membrane proteomes of short- and long-stored CFSE^lo^ subsets.

A physical interaction network (Figure 4C) illustrates potential interactions between the overrepresented proteins in CFSE^hi^ RBCs (95 of 96 proteins). Three protein families were predominantly affected: proteostasis (44%), metabolism and regulations (29%), and vesiculation and trafficking (18%). The increased levels of 95 proteins in long-stored CFSE^hi^ membranes, along with their stable proteome at the intact RBC level, suggests that these proteins were relocated to the plasma membrane in this subset. This hypothesis was confirmed by a second, independent proteomic analysis, showing decreased cytosolic and increased membrane levels of proteins from these 3 protein families in long-stored CFSE^hi^ RBCs (vs. long-stored CFSE^lo^ RBCs, Supplemental Figures 5 and 6).

The most represented family (i.e., proteostasis) comprises 18 proteins involved in protein degradation, including most 19S proteasome subunit proteins, 17 proteins involved in protein folding, and 7 ubiquitin interacting proteins. Quantitative analysis of copy numbers from intact RBC and membrane proteomics data for proteostasis proteins revealed massive relocation to the membrane of long-stored CFSE^hi^ RBCs of most 19S proteasome proteins (mean ± SEM; 77% ± 29%; Supplemental Figure 7A), whereas 20S and 11S proteasome proteins showed minimal relocation (5% ± 3% and 9% ± 1%, respectively; Supplemental Figure 7, B and C, respectively). Ubiquitin-interacting proteins showed variable membrane relocation, reaching more than 50% for 3 proteins (ATG7, UBN4, UBXN1; Supplemental Figure 7D). Most HSP60 and HSP40 chaperone proteins also showed marked membrane relocation (30% ± 8% and 34% ± 27%; Supplemental Figure 8, A and B, respectively), whereas selected proteins among the HSP70, HSP90, and other chaperone families exhibited slight membrane relocation in long-stored CFSE^hi^ RBCs (Supplemental Figure 8, C–E). Taken together, these observations implicate the inability of SMEs to maintain proper energy and redox metabolism during aging in vitro and also suggest that proteostasis is impaired in these cells.

### Proteasomal degradation capability decreases during RBC storage, especially in CFSE^hi^ RBCs.

**Figure 5:**
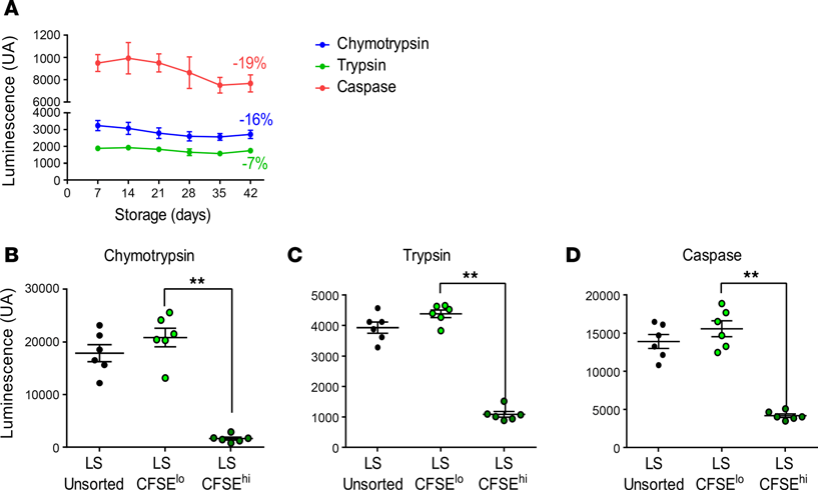
Proteasomal degradation capacity decreases during storage, especially in CFSE^hi^ RBCs. (**A**) Weekly quantification, at the whole-population level, of the chymotrypsin-like (blue curve), trypsin-like (green curve), and caspase-like (red curve) proteasome activities during storage of RBC concentrates in SAGM solution for 42 days (mean ± SEM of 7 RBC concentrates). (**B**–**D**) Chymotrypsin-like (**B**), trypsin-like (**C**), and caspase-like (**D**) activities were measured on subsets for CFSE-stained long-stored unsorted (LS-unsorted) and flow-sorted CFSE^lo^ (LS-CFSE^lo^) and CFSE^hi^ (LS-CFSE^hi^), RBCs. In **A**, a 2-way ANOVA followed by Dunnett’s multiple comparison test was performed, and no significant difference was observed. In **B**–**D**, data are represented as individual points with mean ± SEM of 6 RBC concentrates and **P < 0.01 by Friedman’s 1-way ANOVA followed by Dunn’s multiple comparison test.

Proteostasis is a protein quality control process that safeguards the cellular proteome by stabilizing correctly folded proteins, refolding misfolded proteins, and degrading oxidized/misfolded proteins (42). Proteasomal degradation of oxidized/misfolded proteins contributes to cell proteostasis; interestingly, in previous studies, RBC cytosolic proteasome activities gradually decreased during storage (43, 44). We confirmed a slight nonsignificant decrease in chymotrypsin-like (16%), trypsin-like (7%), and caspase-like activities (19%) of the proteasome during storage (Figure 5A). More importantly, by comparing long-stored CFSE^hi^ and CFSE^lo^ RBCs, significant decreases in chymotrypsin-like (92%, *P* < 0.01), trypsin-like (75%, *P* < 0.01), and caspase-like (73%, *P* < 0.01) proteasome activities were identified in the CFSE^hi^ subset (Figure 5, B–D). In contrast, long-stored and short-stored CFSE^lo^ RBCs had similar proteasome activity levels (Supplemental Figure 9). Taken together, these results identify impaired proteasome degradation capability in SMEs, confirming altered proteostasis function in this morphologically altered subset that increases during aging in vitro.

### Storage lesions occurring during aging in vitro are concentrated in long-stored CFSE^hi^ RBCs, which are preferentially cleared during ex vivo human spleen perfusion.

**Figure 6:**
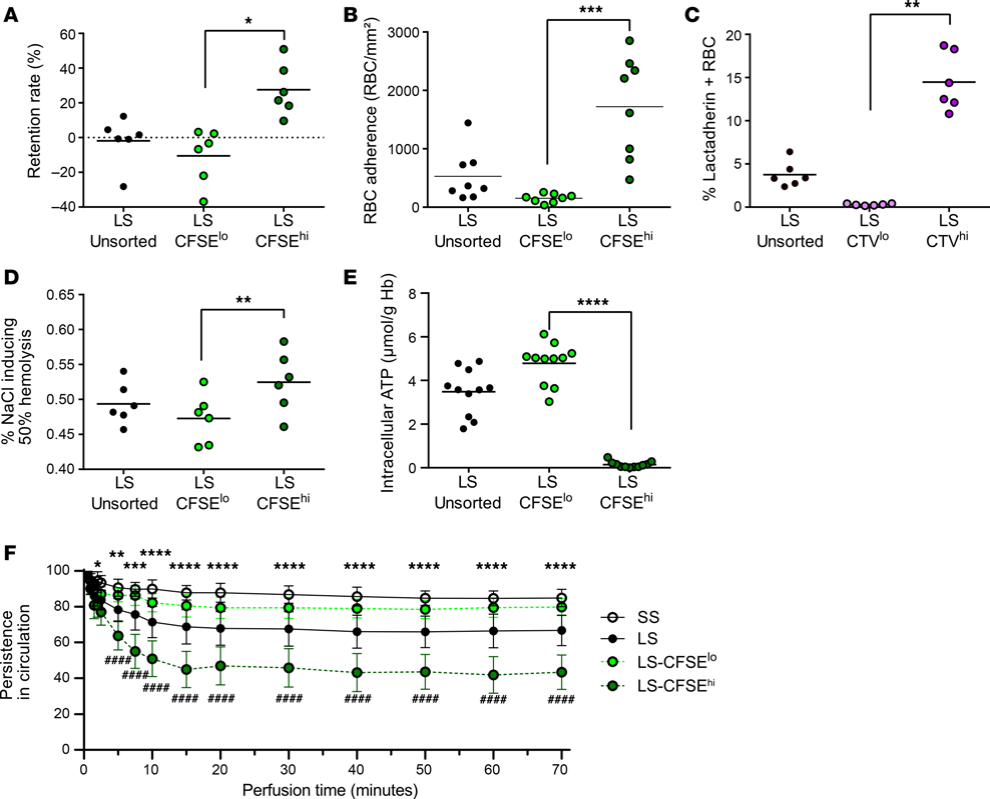
Storage lesions occurring during aging in vitro are concentrated in the long-stored CFSE^hi^ RBC subset; these are preferentially cleared during ex vivo human spleen perfusion. (**A**) Retention rate by microsphiltration, (**B**) dynamic RBC adhesion to endothelial cells, (**C**) RBC surface PS exposure quantified by lactadherin staining, (**D**) osmotic fragility determined by measuring the NaCl concentration required to induce 50% hemolysis, (**E**) intracellular ATP levels, normalized to hemoglobin content, measured with CFSE-stained unsorted long-stored RBCs (LS-unsorted) RBCs and on flow-sorted LS-CFSE^lo^ and LS-CFSE^hi^ RBCs. In **A**–**E**, data are represented as individual points with mean ± SEM of ≥6 RBC concentrates). *P < 0.05, **P < 0.01, ***P < 0.001, ****P < 0.0001 by Friedman’s 1-way ANOVA followed by Dunn’s multiple comparison test. In **C**, CTV-stained RBCs were analyzed to allow lactadherin-FITC staining. (**F**) Kinetics (mean ± SEM of 4 independent perfusions) of the normalized circulating concentrations of stained short-stored (SS), long-stored (LS), LS-CFSE^lo^, and LS-CFSE^hi^ RBC subsets during ex vivo perfusion of human spleen. In **F**, a 2-way ANOVA followed by Šidák’s multiple comparison test was performed. *P < 0.05, **P < 0.01, ***P < 0.001, ****P < 0.0001, and ^####^*P* < 0.0001 by a 2-way ANOVA followed by Šidák’s multiple comparison test. Different symbols are used to show statistical significance: asterisks are used for comparisons of SS vs. LS and pound signs are used for comparisons of LS-CFSE^lo^ vs. LS-CFSE^hi^. No statistically significant differences were seen when comparing SS-CFSE^lo^ vs. LS-CFSE^lo^.

Storage lesion cell biological characteristics were compared between long-stored CFSE^hi^ and CFSE^lo^ subsets. Their deformability was evaluated by microsphiltration, an in vitro spleen-mimicking device (45). The mean retention rate of CFSE^hi^ RBCs was significantly increased (27.5%), as compared with CFSE^lo^ RBCs (–10.6%), indicating reduced deformability of CFSE^hi^ RBCs (*P* < 0.05, Figure 6A). Dynamic endothelial cell adhesion experiments showed a significant 11-fold increase in adherence of long-stored CFSE^hi^ RBCs (1,720 RBC/cm²), as compared with CFSE^lo^ RBCs (154 RBC/cm², *P* < 0.001, Figure 6B). PS-exposing RBCs were identified by lactadherin binding. Since lactadherin-FITC detection by flow cytometry is not compatible with CFSE staining, CellTrace Violet (CTV) staining was used to sort CFSE^hi^ and CFSE^lo^ subsets using a different flow cytometry channel (Supplemental Figure 10). The proportion of PS-exposing long-stored RBCs in the CTV^hi^ subset (14.5%) was increased, as compared with the CTV^lo^ subset (0.3%, *P* < 0.01, Figure 6C). Resistance to osmotic hemolysis also decreased in long-stored CFSE^hi^ RBCs (0.52%), as compared with CFSE^lo^ RBCs (0.47%, *P* < 0.01, Figure 6D). Finally, intracellular ATP levels in long-stored CFSE^hi^ RBCs were very low (0.12 μmol/g Hb), and significantly lower (*P* < 0.0001) than in CFSE^lo^ RBCs (4.69 μmol/g Hb, Figure 6E). Interestingly, long-stored and short-stored CFSE^lo^ RBCs had similar intracellular ATP levels (4.69 vs. 5.58 μmol/g Hb, respectively; Supplemental Figure 11). The lower intracellular ATP levels in long-stored CFSE^hi^ RBCs could be due to a subset-specific decrease in intracellular pH, which would slow down glycolysis. However, experiments using a pH-sensitive probe did not support this hypothesis, because intracellular pH was found to be very similar in CTV^hi^ and CTV^lo^ subsets (Supplemental Figure 12). Taken together, these results show that, although we observed similar intracellular pH levels in all long-stored RBCs, the other RBC storage lesions during aging in vitro are concentrated in the SME subset.

Finally, we compared the circulatory capability of long-stored RBCs mixed with short-stored RBCs using ex vivo perfusion of human spleens (4 independent experiments, Figure 6F). The mean proportion of circulating short-stored and long-stored RBCs decreased by 15% and 33%, respectively, over 70 minutes. Long-stored RBC circulation capacity was significantly decreased at 2 minutes (*P* < 0.05) and at all time points analyzed up until the end of perfusion (70 minutes, *P* < 0.0001), when compared with short-stored RBCs. Among long-stored RBCs, the 2 CFSE subsets had very different profiles of circulatory persistence, with the mean proportion of CFSE^hi^ RBCs decreasing by 57%, whereas that of CFSE^lo^ RBCs decreased by only 20%. Long-stored CFSE^hi^ RBC circulation capacity was significantly decreased at 5 minutes (*P* < 0.0001) and at all time points analyzed up until the end of perfusion (70 minutes, *P* < 0.0001), when compared with long-stored CFSE^lo^ RBCs; the results with the latter subset were similar to those found with short-stored RBCs (not significant). These results confirm that short-stored and long-stored morphologically normal CFSE^lo^ RBCs are similar in their capability to circulate in this ex vivo perfusion model of transfusion. They also suggest that the molecular and cellular alterations of SMEs that were identified herein induce their clearance in this ex vivo transfusion model.

## Discussion

The components of the RBC storage lesion arising during aging in vitro are typically assessed at the whole-population level; as such, any particular result is averaged out over the entire population of RBCs in a given volunteer donor unit. By employing a staining protocol that specifically sorts long-stored morphologically altered RBCs from morphologically normal RBCs, we showed that morphologically altered CFSE^hi^ RBCs are mainly SMEs, which progressively accumulate during storage and are preferentially retained in the spleen. These findings support previous observations positively correlating posttransfusion clearance of SMEs in a mouse model and negatively correlating their posttransfusion recovery in healthy human volunteers (40). Molecularly, marked changes in energy, redox, and lipid-repair metabolism mainly occurred in these morphologically altered RBCs. Additionally, these morphologically altered RBCs exhibited distinct profiles of proteins that were reversibly oxidized, irreversibly oxidized, and relocated to the membrane (including chaperones and most proteins of the 19S proteasome subunit), accompanied by decreased proteasomal enzymatic activity. At the cellular level, characteristics typically associated with RBC clearance were found with the morphologically altered RBCs. Conversely, cellular and molecular properties, and lack of splenic retention ex vivo, of long-stored morphologically normal RBCs resembled those of short-stored RBCs. Taken together, these findings strongly support the concept that the storage lesion differentially affects individual RBCs, revealing biological networks that are overwhelmed by metabolic and oxidative stresses in a distinct RBC subset particularly sensitive to the in vitro aging process.

Our enzymatic assay and metabolomics data confirm the well-documented decrease in intracellular ATP levels during RBC storage (7–9, 46, 47) and further document that the morphologically altered RBCs are more severely affected than morphologically normal RBCs. ATP is required by numerous enzymes, including flippases that internalize PS and membrane pumps that maintain cellular homeostasis; thus, ATP is essential for RBC survival (48–51). In addition, ATP levels negatively correlate with posttransfusion recovery (8, 36, 37, 39, 52), and its subset-specific depletion in SMEs could trigger a cascade of events inducing senescence and clearance.

Depleted pentose phosphates and GAPD metabolites in SMEs identify subset-specific dysregulation of the pentose phosphate pathway (already observed at the whole-population level) (2, 17, 53), suggesting that NADPH levels are inadequate to support multiple antioxidant pathways (54–56), including recycling oxidized glutathione to its reduced form by glutathione reductase. Interestingly, we identified subset-specific depletion of GSH and its precursor (Cys-Gly), and massive increases in oxidized glutathione, suggesting substantial GSH consumption by redox-sensitive thiols (57, 58). Because glutathione synthesis is also ATP-dependent, depleting this important antioxidant system (59, 60) hampers the resistance of SMEs to storage-induced oxidative stress.

Depletion of acylcarnitine metabolites also suggests that the mechanisms required to repair oxidatively damaged membrane lipids are impaired in SMEs. Indeed, lipid repair through the Lands cycle is fueled by the acylcarnitine pool in RBCs (61). Interestingly, impaired lipid repair mechanisms are also observed in sickle cell disease (62). Furthermore, genetic control of carnitine metabolism was recently shown to determine hemolytic propensity during aging in vitro and in vivo, and supplementing stored mouse RBCs with L-carnitine, but not D-carnitine, increased posttransfusion recovery, suggesting that this strategy could improve human RBC storage and transfusion quality (63).

As RBCs age during storage, and protective systems fail, oxidant stress induces protein oxidations that are initially reversible and then become irreversible, such as with carbonylation (1, 6, 13, 15, 16), and with β-elimination of cysteine thiol groups that generate dehydroalanine (17, 58). Our data confirm storage-dependent reversible oxidation in both SMEs and morphologically normal long-stored RBCs, as compared with short-stored RBCs. Irreversible β-elimination of thiol groups was mainly detected in SMEs, supporting the finding of decreased glutathione antioxidant protection in this subset that is particularly sensitive to the in vitro aging process.

Proteomics revealed massive relocation of cytosolic proteins to the membrane of morphologically altered RBCs. Oxidized/misfolded proteins tend to form cytotoxic hydrophobic aggregates, removing them from the pool of active molecules, thereby reducing their function (64). Due to their ability to recognize and bind misfolded/oxidized proteins, proteostasis components are particularly prone to becoming sequestered in aggregates. In this case, they are substantially depleted from the soluble, active pool, negatively impacting the proteostasis network by limiting its capacity, thereby contributing to a vicious circle leading to further aggregate accumulation (65–69). This is particularly relevant for mature RBCs, which no longer have the ability to synthesize new proteins de novo. Our observation that proteostasis proteins relocate to the RBC membrane strongly supports the concept that proteostasis functions are hampered in the morphologically altered RBCs.

Oxidized proteins are susceptible to ATP-independent proteasomal degradation or ubiquitination by ubiquitin-interacting proteins, which then target them for ATP-dependent proteasomal degradation (70, 71). Our results show that proteasomal enzymatic activity is decreased in SMEs, suggesting that the mild decrease in proteasome activity observed during storage (at the whole-population level) (43, 44) is due to important, subset-specific decreases in SMEs. Decreased proteasome activity could be due to ATP depletion, proteasome component relocation to the membrane and/or release in vesicles (43, 44), and/or inefficient unfolding of oxidized/misfolded proteins, preventing their access to the proteasome catalytic core (15, 72).

The relocation of proteins and protein aggregates to the RBC membrane induces irreversible shedding of RBC cytosolic and membrane constituents by vesiculation, resulting in the formation of SMEs through membrane loss and leading to the accumulation of microparticles in storage bag supernatant (73–76). Thus, loss of proteostasis function, accompanied by vesiculation, is consistent with the RBC subset-specific morphological alterations that produce SMEs. The cellular alterations of the morphologically altered RBCs observed herein, including increased PS exposure (a prophagocytic “eat me” signal) (77), endothelial cell adhesion, retention in a spleen-mimetic filter (consistent with diminished deformability from a lower surface/volume ratio) (78, 79), and osmotic fragility (80), could all contribute to RBC retention in the ex vivo human spleen perfusion model (40, 81).

Our data show that only a subset of RBCs is severely altered during storage; the available literature suggests that this corresponds to the older RBCs collected at donation (82, 83). Therefore, our data support the concept that the RBC storage lesion is a form of aging in vitro that enhances aging processes already underway in vivo. For example, during aging in vivo, low ATP levels are seen in senescent RBCs, and, because redox capacity depends upon energy metabolism, they may be more vulnerable to oxidative stresses encountered during refrigerated storage (84, 85). In addition, proteostasis dysfunction is a known hallmark of aging nucleated cells; we now propose that it is also important in aging of nucleus- and mitochondria-free RBCs (42). Finally, in the context of protein oxidation, because mature, organelle-free RBCs can no longer synthesize new proteins, their only alternatives for handling misfolded/oxidized proteins are to protect against (e.g., using ATP, pentose phosphate pathway, GSH), repair (e.g., using chaperones), destroy (e.g., using ubiquitin interacting proteins and proteasomes), or sacrifice (e.g., by vesiculation) them (86).

**Figure 7:**
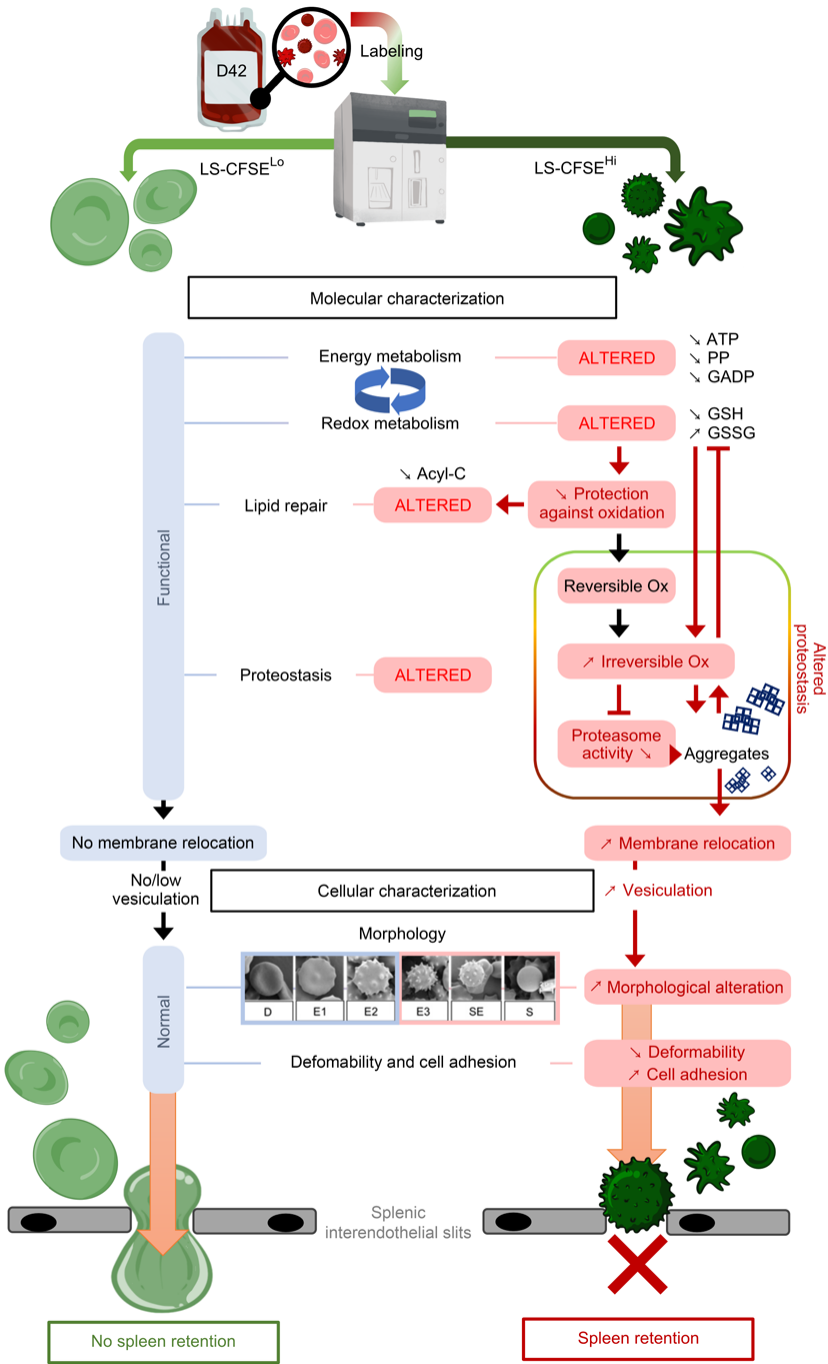
A proposed model to illustrate the main alterations affecting each RBC subset during storage. The main results of the comparative molecular and cellular characterizations (black boxes) of long-stored CFSE^lo^ (light green, discocytes) and long-stored CFSE^hi^ (dark green, echinocytes III, spheroechinocytes, and spherocytes) RBCs. In long-stored CFSE^lo^ RBCs (left), functional energy and redox metabolism sustains effective lipid-repair and proteostasis functions, limiting protein aggregation, membrane relocation, and vesiculation. Functional molecular properties contribute to maintaining normal cellular properties (e.g., morphology, deformability, endothelial cell adhesion) and, thus, their ability to circulate. In long-stored CFSE^hi^ RBCs (right), decreased energy metabolism (e.g., ATP, PP, GAPD) is unable to fuel the redox system effectively, leading to decreased GSH and increased GSSG levels, culminating in dysfunction in lipid repair and proteostasis. The accumulation of irreversibly oxidized proteins could also lead to decreased proteasome function. The combined effect of oxidized protein accumulation and decreased proteasome degradation could fuel a negative feedback loop that further inhibits the redox system, thereby favoring production of toxic protein aggregates, protein relocation to the RBC membrane, and, ultimately, vesiculation. Vesiculation alters morphology, leading to decreased deformability, increased endothelial cell adhesion, and splenic retention. Mild (light blue boxes) and marked alterations are shown (pink boxes); black and red lines represent normal and negative interactions, respectively. PP, pentose phosphate, GADP, DL-GAPDH; GSH, reduced glutathione; GSSG, oxidized glutathione; Acyl-C, acyl-carnitines; Reversible/Irreversible Ox, reversible/irreversible oxidation.

In this conceptual framework, our metabolomics, redox proteomics, proteomics, proteasome activity, and cell biological data are all consistent with a progressive loss of proteostasis function in the older RBCs present “in the bag” at the time of blood donation, leading to the accumulation of SMEs during storage, which are then cleared after transfusion, predominantly by the recipient’s spleen; these results strongly suggest that proteostasis dysfunction contributes to RBC clearance (Figure 7).

Nonetheless, our study has some limitations. For example, the staining protocols require a 6-hour minimum incubation time at 37°C to discriminate between CFSE^hi^ and CFSE^lo^ subsets. The observed differences may reflect end-of-storage RBC properties, which are normally evaluated directly following storage at 4°C, and/or RBC properties similar to those described in prior in vitro transfusion models (87, 88); thus, the observed phenotype may be exacerbated by the 37°C incubation. Despite potential effects of the staining/sorting protocol, the short-stored CFSE^lo^ and long-stored CFSE^hi^ and CFSE^lo^ subsets experienced the same treatment and, therefore, are directly comparable. As other limitations, omics experiments were conducted on a limited number of random donors, sex as a variable was not considered, and blood type, age, and biological sex were the only personal data collected from these donors. Additional donors and the collection of additional donor information would be needed to verify whether donor factors determine the subset-specific metabolic and proteostasis dysfunctions observed in this study and the proportion of morphologically altered RBCs that accumulate during storage. In addition, regarding the omics data, the measured metabolites and proteins represent only a snapshot of cellular activity; performing dynamic flux experiments and measuring functional activities of key proteins (e.g., the glucose transporter, phosphofructokinase, glucose-6-phosphate dehydrogenase) would confirm and extend the current observations.

This study has important implications in transfusion medicine. Our observation that the metabolic dysfunction occurs primarily in the morphologically altered RBC subset is consistent with studies reporting that metabolic (e.g., ATP, acyl-carnitine, hypoxanthine) and morphologic (e.g., morphology index, SMEs) markers can be used to predict transfusion recovery (8, 38–40, 63, 89).

In Europe, hemolysis is the only in vitro marker of storage quality required for any new process involved in preparing and storing RBC concentrates, and it must be <0.8% at the end-of-storage time point (90). However, hemolysis in vitro does not correlate with posttransfusion recovery in vivo; therefore, it does not directly evaluate the RBC properties that determine transfusion efficacy (91). In the United States, in addition to hemolysis in vitro (which must be <1.0% at the end-of-storage time point), an average 51-chromium–labeled RBC posttransfusion recovery of >75% at the end-of-storage time point in healthy volunteers receiving an autologous transfusion is required for FDA approval; nonetheless, this expensive and technically challenging measure, which is only performed at a few centers, does provide information on stored RBC circulatory capacity (92).

Our results show that subset-specific proteostasis and metabolic dysfunction characterize the morphologically altered RBCs that accumulate during storage and that are preferentially retained in the spleen following transfusion. Their rapid posttransfusion clearance likely explains the decreased Hb increment seen with transfusion of long-stored RBCs (25, 34, 35). Quantifying them in RBC concentrates by either imaging flow cytometry (i.e., SMEs) or flow cytometry (i.e., CFSE^hi^ RBCs) can provide an in vitro cellular marker that directly measures the RBC subset with decreased posttransfusion circulation capacity; this approach could be used, along with measuring end-of-storage hemolysis in vitro, to evaluate new manufacturing processes (e.g., hypoxic, ref. 39; pathogen-inactivated, ref. 93; and DEHP-free storage, ref. 94) and storage alternatives (e.g., AS-7, ref. 95). It also has the potential to complement hemolysis markers that are already used with omics approaches to identify donor factors that affect RBC storage and transfusion quality (25, 34, 96), paving the way for precision (i.e., personalized) transfusion medicine (97, 98).

Refrigerated storage of organelle-free, Hb-rich RBCs has historically been regarded as a useful model of oxidant stress biology and cellular aging. The current results confirm these assumptions and suggest that it may also provide a model of proteostasis dysfunction. Additional exploration of proteostasis during RBC storage, and in physiologic and pathophysiologic contexts, could further deepen our understanding of RBC aging in vitro and in vivo.

## Methods

Detailed information is provided in Supplemental Methods.

### Sex as a biological variable.

RBCs and spleens used in this study are derived from anonymized human samples. Sex was not considered as a biological variable.

### RBC concentrate collection and storage.

Leukoreduced RBC concentrates from healthy donors were obtained from the Etablissement Français du Sang and stored in SAGM at 2°C–6°C for 44 days. Samples were aseptically collected and analyzed on storage days 3–10 (short-stored) and 40–44 (long-stored). Donor characteristics (age, biological sex, blood type) can be found in Supplemental Tables 2–8.

### CFDA-SE and CTV staining.

RBCs were washed and stained with CFDA-SE for 20 minutes at 37°C (5.5 million RBCs/mL, 0.05 μM CFDA-SE in PBS). CFDA-SE (nonfluorescent) diffuses passively into cells and is processed rapidly by cellular esterases, resulting in highly fluorescent CFSEs that bind cellular components. Stained RBCs were then washed, suspended in RPMIc (i.e., RPMI 1640 supplemented with 10% FBS and 1% antibiotic/antimycotic solution), and incubated overnight at 37°C. Following the overnight incubation, a subset of cells with decreased CFSE fluorescence intensity (identified as CFSE^lo^ RBCs) and a subset with CFSE fluorescence intensity similar to cells immediately after staining (CFSE^hi^ RBCs) could be observed by flow cytometry. These stained/incubated RBCs were washed, resuspended in fresh RPMIc, and stored at 4°C until analysis. When needed, a CTV staining (1 μM) was performed using the same protocol, also yielding a RBC subset with decreased fluorescence intensity (CTV^lo^) and a RBC subset with CTV fluorescence intensity similar to cells immediately after staining (CTV^hi^ RBCs).

### Cell sorting.

CFSE^lo^ and CFSE^hi^ RBCs were sorted using a MA900 Cell Sorter (Sony). Sorted RBCs were centrifuged after collection, resuspended in RPMIc, and stored at 4°C until analysis or frozen (for omics experiments). As controls, stained RBCs were sorted using only size/structure parameters (unsorted condition).

### Imaging flow cytometry.

Imaging flow cytometry (ImageStream X Mark II; Amnis Flow Cytometry, Luminex) examined RBC dimensions and morphology, as described previously (19). Bright-field images (×60 magnification) were analyzed using dedicated computer software (IDEAS [version 6.2]; Amnis) to determine the proportions of SMEs.

### Scanning electron microscopy.

RBCs were prepared as described previously (40) and observed using a Zeiss Ultra plus field emission-scanning electron microscope.

### Metabolomics and redox-proteomics.

Metabolomics were performed, as described previously (99). Redox-proteomics were performed using Filter Aided Sample Preparation digestion and nano ultra-high-pressure liquid chromatography tandem mass spectrometry (TIMS TOF Pro 2 Single Cell Proteomics, Bruker Daltonics) (17, 100).

### Proteomics of intact RBCs and RBC membranes.

Proteomics of intact RBCs and RBC membranes (i.e., ghosts) were performed by nanoscale liquid chromatography coupled to tandem mass spectrometry (Dionex U3000 RSLC, Thermo Fisher Scientific), as described previously (101).

### PS exposure.

The number of RBCs exposing PS were quantified using FITC-conjugated bovine lactadherin (lactadherin-FITC, Cryopep), as described previously (9).

### Microsphiltration.

Microsphiltration plates containing calibrated metal microspheres (to mimic spleen interendothelial slits) were used to assess RBC deformability, as described previously (9). Diluent RBCs (95%) were stained with CellTrace Far Red (Life Technologies), test RBCs (5%) were sorted CFSE^lo^ and CFSE^hi^ subsets, and upstream mixtures were prepared at a 1% hematocrit in Krebs-albumin solution, filtered through the microsphere layer, and washed twice. Downstream suspensions were then collected. Upstream and downstream proportions of test RBCs were evaluated by flow cytometry, and retention rates were calculated as follows: [(UP – DW)/UP] × 100, where UP represents the percentage of test RBCs in upstream sample and DW denotes the percentage of test RBCs in downstream sample.

### ATP.

Intracellular ATP concentrations were determined using an ATP assay kit (ATPlite, PerkinElmer) and normalized against the Hb concentration of each sample (μmol/g Hb).

### Osmotic fragility.

RBC osmotic fragility was determined, as described previously (19), using 3,3′, 5,5′′-tetramethylbenzidine to increase sensitivity.

### Dynamic RBC adhesion to endothelial cells.

Dynamic adhesion experiments were performed, as described previously (9). Briefly, human microvascular endothelial cell line 1 (HMEC-1, ATCC-CRL-3243) cells were cultured in microchannels, and RBCs were perfused (10 minutes, 0.2 dyn/cm^2^). The shear stress was increased every 5 minutes (0.5 and 1 dyn/cm^2^) to remove less adherent RBCs. Bright-field images at 1 dyn/cm² were analyzed to quantify adherent RBCs.

### Proteasome activity.

Proteasome-specific activities (i.e., chymotrypsin-like, trypsin-like, caspase-like) were measured by using Cell-Based Proteasome-Glo Assays (Promega), following the manufacturer’s recommendations.

### Human spleen retrieval and ex vivo perfusion.

Spleens (macroscopically and microscopically normal) were retrieved and processed, as described previously (102), from patients undergoing distal splenopancreatectomy for pancreatic disease. The main splenic artery was cannulated and spleens were flushed with cold Krebs-albumin solution. Spleens were perfused with long-stored CFSE-stained RBCs and Celltrace Far Red-stained (CTFR, Life Technologies) short-stored RBCs (final hematocrit of 5%–30% in Krebs-albumin solution) for 70 minutes at 37°C. During RBC perfusion, arterial pressure was maintained at 60–120 mm Hg, perfusate flow was set at 1 mL/g spleen/min, and the temperature of the spleen capsule was maintained at 37°C. In addition, key parameters (e.g., pH, HCO3^–^, glucose, lactate) were closely monitored, and supplemental perfusion solution (e.g., glucose, bicarbonates) was added when necessary. Samples were retrieved from the circuit for flow cytometric quantification. Persistence in circulation was calculated as follows: (percentage of stained RBCs in sample/percentage of stained RBCs at T0) × 100.

### Statistics.

Data were analyzed using GraphPad Prism version 9.2.0 for Windows. All data with >8 samples were tested for normality with the D’Agostino and Pearson test. One- and two-way ANOVA with Šidák’s multiple comparison test was performed for parametric data. ANOVA of Friedman’s test with Dunn’s multiple comparison test was performed for nonparametric data. Two-tailed Student’s *t* test was performed when 2 groups were compared. A *P* value of less than 0.05 was considered statistically significant.

### Study approval.

The study was conducted according to the Declaration of Helsinki. Human spleens were retrieved in the context of the Spleenvivo project approved by the “Ile-de-France II” Institutional Review Board (Hôpital Necker, Paris, France) on September 4, 2017 (#CPP 2015-02-05 SM2 DC). Written informed consent was received prior to participation.

### Data availability.

The values for all data points in graphs are reported in the Supporting Data Values file. Mass spectrometry proteomics raw data were deposited in the ProteomeXchange Consortium via the PRIDE (103) partner repository, with dataset identifier PXD049411 (https://www.ebi.ac.uk/pride/archive/projects/PXD049411).

## Author contributions

SP, MM, M Dussiot, LL, YH, AF, CR, PAN, MKR, M Dzieciatkowska, JB, SG, and AS performed experiments. FP and SD provided human spleens. SP, MM, M Dussiot, EFG, AD, and PA analyzed the data. SP, MM, and M Dussiot prepared figures. MC, SP, MM, CR, SLS, OH, PAB, AD, and PA designed the research. SP, MM, M Dussiot, and PA wrote the manuscript. CR, EFG, SLS, PAB, and AD edited the manuscript. All the authors critically contributed to the progress of the project and finalization of this manuscript. The order of first authors reflects the contribution to writing and editing of the manuscript.

## Supplementary Material

Supplemental data

Supporting data values

## Version 1 03/11/2025

In-Press Preview

## Version 2 05/01/2025

Electronic publication
