# Supplementary material for: Proteostasis and metabolic dysfunction characterize a subset of storage-induced senescent erythrocytes targeted for posttransfusion clearance
Source: J Clin Invest. 2025 Mar 11;135(9):e183099. doi: 10.1172/JCI183099 (PMC12043093; doi:10.1172/JCI183099)
Supplement: Supplemental data [file jci-135-183099-s035.pdf]

Supplemental Data

Methods

Cell sorting

Sorting of $CFSE^{low}$ and $CFSE^{high}$ RBCs was performed using a MA900 Cell Sorter (Sony) with a 100µm sorting-chip at the maximum speed of 10,000 events per second in semi-purity mode. Sorted RBCs were collected in tubes containing 1mL of RPMIc, then centrifuged, resuspended in RPMIc, and stored at 4°C until analysis. Stained RBCs that were sorted based only on size/structure parameters were used as controls (unsorted condition).

Imaging flow cytometry (IFC) analysis

IFC was performed with an ImageStream X Mark II (Amnis® Flow Cytometry, Luminex, Seattle, WA, USA) to determine RBC morphology (1). Just before acquisition, RBCs were suspended at 1% hematocrit in Krebs-albumin solution (Krebs-Henseleit buffer, Sigma-Aldrich) modified with 2g/L of glucose, 2.1g/L of sodium bicarbonate, 0.175g/L of calcium chloride dihydrate, and 5g/L of lipid-rich bovine serum albumin (Albu-MAX II, Life Technologies). Images (x60 magnification) were recorded (INSPIRE software, AMNIS) using the brightfield channel to be processed using dedicated computer software (IDEAS [version 6.2]; Amnis). Focused cells and single cells were respectively selected using the features gradient RMS_M01_Ch01 and Aspect ratio_M01_Ch01 versus Area_M01_Ch01. Front views were selected using the feature Circularity_Object (M01, Ch01, Tight) and projected surface area was determined using the feature Area_Object (M01, Ch01, Tight). At least 6000 front views of focused single RBCs per condition were analyzed. The SME proportion was determined independently for each donor, using the nadir of the bimodal frequency histograms as the gating boundary.

Proteomics of intact RBCs and RBC ghosts

Proteomics of intact RBCs and RBC ghosts were performed by nanoscale liquid chromatography coupled to tandem mass spectrometry (nLC-MS/MS). The mass spectrometry proteomics data were deposited in the ProteomeXchange Consortium via the PRIDE (2) partner repository with the dataset identifier PXD049411.

Sample processing: To prepare ghosts, hypotonic buffer was used to lyse RBCs: 5mM Na_2HPO_4, 0.35mM EDTA, 1mM phenylmethylsulfonyl fluoride (PMSF). Cell membranes were pelleted by centrifugation for 20 minutes at 15600 x *g*. The ghost pellet was washed several times with hypotonic buffer to obtain a white pellet. Five million RBCs and 10 million ghosts per sample were boiled in lysis buffer (50mM TRIS pH 8.5 with 2% SDS) for 5 minutes at 95°C. Protein concentrations were determined using a bicinchoninic acid assay (BCA kit; Pierce). Disulfide bridges from 50 micrograms of protein for intact RBC samples, and from the totality of proteins for ghost samples, were reduced using 10mM tris(2-carboxyethyl)phosphine; the resulting free thiols were protected using 50mM chloroacetamide for 5 minutes at 95°C. Proteins were trypsin-digested overnight using the filtered-aided-sample-preparation method, as described (3). Eluted peptides were separated into 5 fractions using strong cation exchange (SCX) StageTips and then vacuum-dried during centrifugation in a Speed Vac (Eppendorf).

nLC-MS/MS proteomics: MS analyses were performed on a Dionex U3000 RSLC nano-LC system coupled to an Orbitrap Fusion mass spectrometer (Thermo Fisher Scientific). Peptides from each SCX fraction were solubilized in 0.1% trifluoracetic acid containing 10% acetonitrile, loaded, concentrated, and washed on a C18 reverse phase precolumn (3-µm particle size, 100 Å pore size, 75-µm inner diameter, 2-cm length; Thermo Fischer Scientific). Peptides were then separated on a C18 column (2-mm particle size, 75-mm inner diameter, 25-

cm length; Thermo Fisher Scientific) with a 3 hour gradient starting from 99% solvent A (0.1% formic acid) and ending with 55% solvent B (80% acetonitrile, 0.085% formic acid). The mass spectrometer acquired data throughout the elution process and operated in a data-dependent scheme with full MS scans acquired with the Orbitrap, followed by as many MS/MS ion trap HCD spectra 3 seconds can fit (data-dependent acquisition with top speed mode: 3 second cycle) using the following settings for full MS: automatic gain control (AGC) target value: $1.10e^{6}$, maximum ion injection time (MIIT): 60 milliseconds, resolution: 6.10e4, m/z range 350–1500. For HCD MS/MS: Quadrupole filtering, Normalised Collision Energy: 30. Ion trap rapid detection: isolation width: 1.6 Th, minimum signal threshold: 5000, AGC: $1.10e^{5}$, MIIT: 60 milliseconds, resolution: $3.10e^{4}$. A dynamic exclusion time was set at 30 seconds. Peptides with charge state less than 1 or greater than 7 were excluded from fragmentation.

Analysis of nLC-MS/MS data: Identifications and quantifications were performed using MaxQuant version 2.0.3.0 (4), the reviewed Human Uniprot database (released March 2020), and a list of frequent contaminant sequences. The false discovery rate was kept below 1% on both peptides and proteins, and a maximum 2 missed cleavages was allowed. Carbamidomethylation of cysteines was set as a constant modification and acetylation of the protein N-terminus and oxidation of methionine were set as variable modifications. Label-free protein quantification (LFQ) was performed using both unique and razor peptides with at least 2 ratio counts. The “match between runs” (MBR) option was allowed with a match time window of 0.7 minutes and an alignment time window of 20 minutes. Absolute quantification of cell proteins for intact RBC proteomes was performed using calculated MCH values, as described (5). For RBC ghost proteome absolute quantifications, calculated BAND3 in the corresponding proteome was used as a reference.

Statistical analysis: Data were imported into the Perseus software version 1.6.15.0 (6). Reverse and contaminant and only identified by site proteins were excluded from analysis. Only

proteins quantified in at least 3 samples of one condition were selected for two samples Student's T-Test.

Metabolomics and redox-proteomics

Metabolomics and redox-proteomics from intact RBCs were performed by ultra-high-pressure liquid chromatography coupled to tandem mass spectrometry (UHPLC-MS/MS).

Sample processing and metabolite extraction: Flow-sorted RBCs were extracted at a concentration of 4 million cells per ml in methanol:acetonitrile:water (5:3:2, *v/v/v*). After vortexing at 4°C for 30 minutes, soluble extracts were separated from the protein pellet by centrifugation for 10 minutes at 18,000 x *g* at 4°C, and stored at −80°C until analysis.

UHPLC-MS/MS metabolomics: Analyses were performed using a Vanquish UHPLC coupled online to a Q Exactive mass spectrometer (Thermo Fisher, Bremen, Germany). Samples were analyzed using a 1 minute and 5 minute gradient, as described (7–9). Solvents were supplemented with 0.1% formic acid for positive mode runs and 1 mM ammonium acetate for negative mode runs. MS acquisition, data analysis, and elaboration were performed, as described.(7–9)

UHPLC-MS/MS redox-proteomics: Proteomics analyses were performed via Filter Aided Sample Preparation digestion and nano-HPLC-MS/MS identification (TIMS TOF Pro 2 Single Cell Proteomics, Bruker Daltonics, Bremen, Germany), as described (10).

Statistical analyses: Graphs and statistical analyses were prepared with MetaboAnalyst 5.0 (11).

Acronyms in Figure 2: ACSL: acyl-CoA synthetase; acyl-CX:Y: acylcarnitine with X:Y acyl chain; CMP-Neu5Ac: CMP-N-acetylneuraminate; CoA: Coenzyme A; CPT: carnitine palmitoyltransferase; Cys-Cys: cystine, two cysteines bonded together; Cys-Gly: cysteinyl-glycine; DHAP: dihydroxyacetone phosphate; FA: fatty acid; G6PD: glucose-6-phosphate

dehydrogenase; GADP: glyceraldehyde 3-phosphate; Glc-6P: glucose 6-phosphate; Glycerol 3P: glycerol 3-phosphate; GSH/GSSG: reduced/oxidized glutathione; GSR: glutathione-disulfide reductase; LPA/PA: lyso/phosphatidic acyl; LPAT: lysophospholipid acyltransferases; NAD+: oxidized nicotinamide adenine dinucleotide; NADPH/NADP+: reduced/oxidized nicotinamide adenine dinucleotide phosphate; Pentose P: Pentose Phosphate, alpha-D-Ribose 1-phosphate was detected here; PL: phospholipid; PLA2: phospholipaseA2; PPP: pentose phosphate pathway; THcHDO: 3D-(3-5/4)-Trihydroxycyclohexane-1-2-dione.

Osmotic fragility

RBC osmotic fragility was determined, as described (1), with modifications to increase sensitivity. Briefly, 0.8 million RBCs were washed in PBS, incubated for 45 minutes in hypotonic NaCl-PO4 solution (equivalent to NaCl solution, ranging from 0% to 0.9%), and centrifuged (800 x *g*, 5 minutes). Heme-mediated (non-HRP) peroxidase activity of the released hemoglobin was revealed by adding 50µL of 3,3’, 5,5”-tetramethylbenzidine (TMB) to 12.5µL of supernatant for 1 hour. Absorbance was measured at 655nm using a spectrophotometer, and the percent hemolysis for each salt concentration was calculated.

Dynamic RBC adhesion on endothelial cells

Human microvascular endothelial cell line 1 (HMEC-1, ATCC-CRL-3243) cells were seeded at 10^8 cells/mL in Vena8 Endothelial+ Biochips (Cellix Ltd, Dublin, Ireland), previously coated with 40µL of 0.2% gelatin in PBS. Cells were then incubated for 2 hours at 37°C, permitting cell attachment, and then cultured for 48 hours using a Kima pump (Cellix Ltd.). RBCs were washed in PBS and resuspended to a 1% hematocrit in Hank’s buffer supplemented with 0.4% bovine albumin, Ca^{2+}, Mg^{2+}, and HEPES (1mM). To initiate adhesion, a first perfusion step was performed (10 minutes, 0.2 dyn/cm2), enabling interactions between RBCs and endothelial cells. Then the shear stress was increased every 5 minutes (0.5 and then 1 dyn/cm2) to remove

less adherent RBCs. Brightfield imaging of adherent RBCs was performed at 10x magnification (AxioObserver Z1, Zeiss). The number of adherent RBCs was determined from 10 pictures taken at the end of the 1 dyn/cm² step for each condition.

Proteasome activity

Proteasome activity was measured using Cell-Based Proteasome-Glo™ Assays (Promega), according to the manufacturer's recommendations. Briefly, 100,000 RBCs were mixed with the specific substrate for each activity (Suc-LLVY, Z-LRR, and Z-nLPnLD for chymotrypsin-like, trypsin-like, and caspase-like activity, respectively), diluted in the detection reagent, and incubated for 10 minutes at RT. Luminescence was read with an Infinite 200 Pro (Tecan).

Intracellular pH measurement

Intracellular pH measures in long-stored CTV-stained RBCs were performed using a pH-sensitive probe, pHrodo green (Life Technologies), according to the manufacturer's recommendations. Briefly, 1 million RBCs were stained with 100 µL of pH staining solution (pHrodo green 1X and PowerLoad concentrate 1X diluted in Live Cell Imaging Solution) for 30 minutes at 37°C. RBCs were then washed and resuspended in RPMIc before IFC acquisition.

Images (x60 magnification) were recorded (INSPIRE software, AMNIS) using the 450-560 (pHrodo green) and 435-505 (CTV) channels to be processed using dedicated computer software (IDEAS [version 6.2]; Amnis). Focused cells and single cells were selected using the features gradient RMS_M01_Ch01 and Aspect ratio_M01_Ch01 versus Area_M01_Ch01, respectively. CTV^{low} and CTV^{high} RBCs were selected using the feature Intensity_Object(M01, 1-BF, Tight)_7-CTV and pHrodo green mean fluorescence intensity was determined using the feature Intensity_Object(M01, 1-BF, Tight)_2-pHrodo green.

Supplemental Tables

Family		Gene Names	Peak area SS $CFSE^{low}$	Peak area LS $CFSE^{low}$	Peak area LS $CFSE^{high}$
Proteostasis (24/83 = 29%)	Proteasome	PSMD2	2.86	5.86	5.43
		PSMD6	0.14	1.57	1.14
		PSMD13	0.29	2.57	2.00
		FBXO7	0.29	3.29	3.29
		CAND1	0.57	2.43	3.14
		UBE2V1	9.71	11.57	17.00
		UBE2V2	1.43	4.00	6.43
		PSMC1	0.71	3.43	2.14
		PSMD1	6.00	10.43	5.43
		PSMC2	0.00	1.57	0.43
		PSMC5	0.14	1.57	0.14
		PSMA5	1.71	2.71	0.86
		PSMB7	0.14	1.57	0.57
		COPS6	1.71	4.00	1.57
		USP9X	0.00	0.71	0.14
		RNF123	1.71	4.71	3.14
	Chaperones	HSPA8	29.43	43.71	38.00
		CCT6A	2.43	5.14	4.57
		STIP1	5.71	10.43	13.43
		HSP90AB1	0.00	0.43	1.14
		NAP1L4	0.43	1.00	1.14
		ST13;ST13P5;S	3.14	8.29	5.43
		CCT2	10.43	19.43	13.71
		CCT4	4.43	10.14	4.57
Cytoskeleton (9/83 = 11%)		ACTB	41.57	51.00	54.71
		SPTA1	107.29	134.00	128.43
		SPTB	129.57	154.43	162.29
		ANK1	60.43	77.00	76.00
		MSN	0.86	4.00	2.00
		JUP	0.00	0.00	1.29
		WDR1	2.86	2.57	4.29
		CFL1	0.00	0.14	1.43
		EPB41	0.00	0.14	1.29
Anti-oxidant (7/83 = 8%)		PRDX1	3.29	5.71	8.86
		PRDX6	12.57	20.29	12.71
		BLVRB	66.14	89.86	81.14
		TXNL1	0.14	1.29	0.57
		CAT	0.00	0.14	2.00
		PARK7	0.00	0.14	1.00
		PRDX2	0.71	3.71	3.43

Family	Gene Names	Peak area SS $CFSE^{low}$	Peak area LS $CFSE^{low}$	Peak area LS $CFSE^{high}$
Glycolysis (5/83 = 6%)	ENO1	5.00	9.00	9.86
	TALDO1	4.71	6.71	5.14
	PGK1	25.14	28.29	32.00
	PFKM	2.00	3.71	5.57
	GAPDH	0.29	2.57	3.71
Transport (5/83 = 6%)	AGO2	1.71	3.57	2.71
	IPO9	0.71	2.29	0.71
	AP2A1	0.00	1.57	0.71
	AP2B1	0.43	1.71	0.57
	AP2M1	0.00	0.86	0.14
Hemoglobins (3/83 = 3.6%)	HBA1	733.00	824.14	886.00
	HBB	266.29	368.43	355.71
	HBD	32.29	39.86	53.14
Other (30/83 = 36%)	ACLY	5.57	11.86	13.71
	ATP2B4	0.00	0.71	0.86
	FABP5	0.29	1.43	2.43
	GDI2	12.86	19.86	20.14
	EIF4A1	9.00	12.29	12.43
	NME1	30.43	37.14	38.43
	NIF3L1	0.29	1.57	1.57
	PAICS	2.14	5.14	4.71
	STOM	5.86	10.43	9.57
	ARG1	0.00	0.14	1.00
	CA1	55.00	71.29	90.29
	DNPEP	0.57	0.29	1.29
	KRT14	8.29	12.57	19.00
	KRT84	0.00	0.00	1.29
	TSTA3	4.00	5.29	6.43
	TPM3	2.29	3.14	4.43
	KRT1	27.00	31.57	37.57
	ATP6V1A	2.00	4.43	2.57
	AARS	0.29	1.86	0.57
	THOP1	0.00	1.71	0.00
	SEC14L2	0.71	2.14	1.14
	PNP	22.43	37.00	25.86
	LTA4H	0.00	2.43	1.57
	MCTS1	0.29	1.71	0.29
	BSG	0.29	1.43	0.57
	ADK	0.00	1.86	0.43
	ANXA7	3.86	9.29	4.86
	YWHAB	2.86	5.00	3.00
	YWHAG	3.86	6.00	1.43
	CD44	0.86	1.14	0.00

Supplemental Table 1: Classification of oxidized proteins during storage. Gene names from the reviewed Human Uniprot database are indicated. Peak areas are indicated for each detected oxidized protein. Proteins denoted in black are reversibly oxidized, whereas those denoted in brown are irreversibly oxidized (Cys to DHA).

Internal donor number	Figure panel	Blood type	Age (y)	Biological sex
245	1A	A+	27	F
246	1A	A-	24	F
247	1A	A+	18	F
248	1A	O+	22	F
249	1A	A-	26	M
250	1A	O+	35	M
251	1A	O-	26	F
252	1A	O+	53	F
1	1B	NA	NA	NA
4	1B	NA	NA	NA
6	1B	NA	NA	NA
8	1B	NA	NA	NA
176	1B	O+	48	F
177	1B	O+	19	M
180	1B	B+	41	F
181	1B	O+	38	F
186	1B	O-	46	F
188	1B	O-	40	F
190	1B	O-	23	F
207	1B	O+	20	F

Supplemental Table 2: Donor characteristics for the morphologically-altered RBCs quantification. Blood type, age, and biological sex of each donor used for the weekly quantification of SMEs and CFSE^{high} RBCs (Figure 1A) and SMEs quantification in the flow-sorted RBCs (Figure 1B).

Internal donor number	Blood type	Age (y)	Biological sex
195	A+	50	M
196	O+	26	F
198	A+	19	F
199	B+	21	F
209	O+	38	F
210	A-	27	F
211	O+	69	F

Supplemental Table 3: Donor characteristics for the first omics experiment. Blood type, age, and biological sex of each donor used for the metabolomics experiment (Figure 2; Supplemental Figure 1; Supplemental Figure 2) and the redox proteomics experiment (Figure 3; Supplemental Table 1) conducted on whole RBCs.

Internal donor number	Blood type	Age (y)	Biological sex
1	NA	NA	NA
4	NA	NA	NA
6	NA	NA	NA
8	NA	NA	NA
194	O+	70	M
195	A+	50	M
203	O+	29	F

Supplemental Table 4: Donor characteristics for the second omics experiment. Blood type, age, and biological sex of each donor used for the proteomics experiment conducted on whole RBCs and membrane preparations (Figure 4; Supplemental Figure 4; Supplemental Figure 7; Supplemental Figure 8).

Internal donor number	Figure panel	Blood type	Age (y)	Biological sex
292	5B-5C-5D	A+	19	M
293	5B-5C-5D	A-	34	M
294	5B-5C-5D	A+	44	M
295	5A-5B-5C-5D	A+	28	F
296	5A-5B-5C-5D	A-	21	F
297	5A-5B-5C-5D	A+	34	F
298	5A	B+	22	F
299	5A	O+	20	M
300	5A	O+	31	F
301	5A	O+	59	F

Supplemental Table 5 : Donor characteristics for the proteasome activity quantification. Blood type, age, and biological sex of each donor used for the weekly quantification of proteasome activity along storage (Figure 5A) and in flow-sorted RBCs (Figure 5B).

Internal donor number	Figure	Blood type	Age (y)	Biological sex
1	6A-6B-6E	NA	NA	NA
4	6A-6B-6E	NA	NA	NA
6	6A-6B-6E	NA	NA	NA
8	6A-6B-6E	NA	NA	NA
174	6B-6E	O+	49	F
175	6A-6B-6E	O+	19	M
176	6B-6E	O+	48	F
177	6B-6E	O+	19	M
180	6A	B+	41	F
191	6D	AB+	33	M
193	6D	A+	51	M
195	6D	A+	50	M
207	6D	O+	20	F
226	6C	A+	NA	NA
227	6C	A+	NA	NA
228	6C	A+	NA	NA
230	6C	A+	43	F
232	6E	O+	21	M
234	6C	A+	60	F
235	6C-6E	O+	24	F
236	6E	O-	19	F
240	6D	B+	45	F
242	6D	A+	53	F

Supplemental Table 6 : Donor characteristics for the storage lesion evaluation in flow-sorted RBCs. Blood type, age, and biological sex of each donor used for retention rate by microsphiltration (Figure 6A), dynamic adhesion to endothelial cells (Figure 6B), RBC surface PS exposure (Figure 6C), osmotic fragility (Figure 6D) and intracellular ATP level (Figure 6E).

Internal donor number	Blood type	Age (y)	Biological sex
177	O+	19	M
183	A+	68	F
186	O-	46	F
254	O+	19	F

Supplemental Table 7 : Donor characteristics for the ex vivo human spleen perfusion experiments. Blood type, age, and biological sex of each donor used for human spleen perfusion experiments (Figure 6F).

Internal donor number	Blood type	Age (y)	Biological sex
247	A+	18	F
248	O+	22	F
249	A-	26	M
251	O-	26	F
252	O+	55	F
253	O+	25	F
254	O+	19	F
255	A+	43	M

Supplemental Table 8: Donor characteristics for the third omics experiment. Blood type, age, and biological sex of each donor used for the redox proteomics confirmation cohort (Supplemental Figure 3) and the proteomics experiment (Supplemental Figure 5; Supplemental Figure 6) conducted on cytosol and membrane preparations.

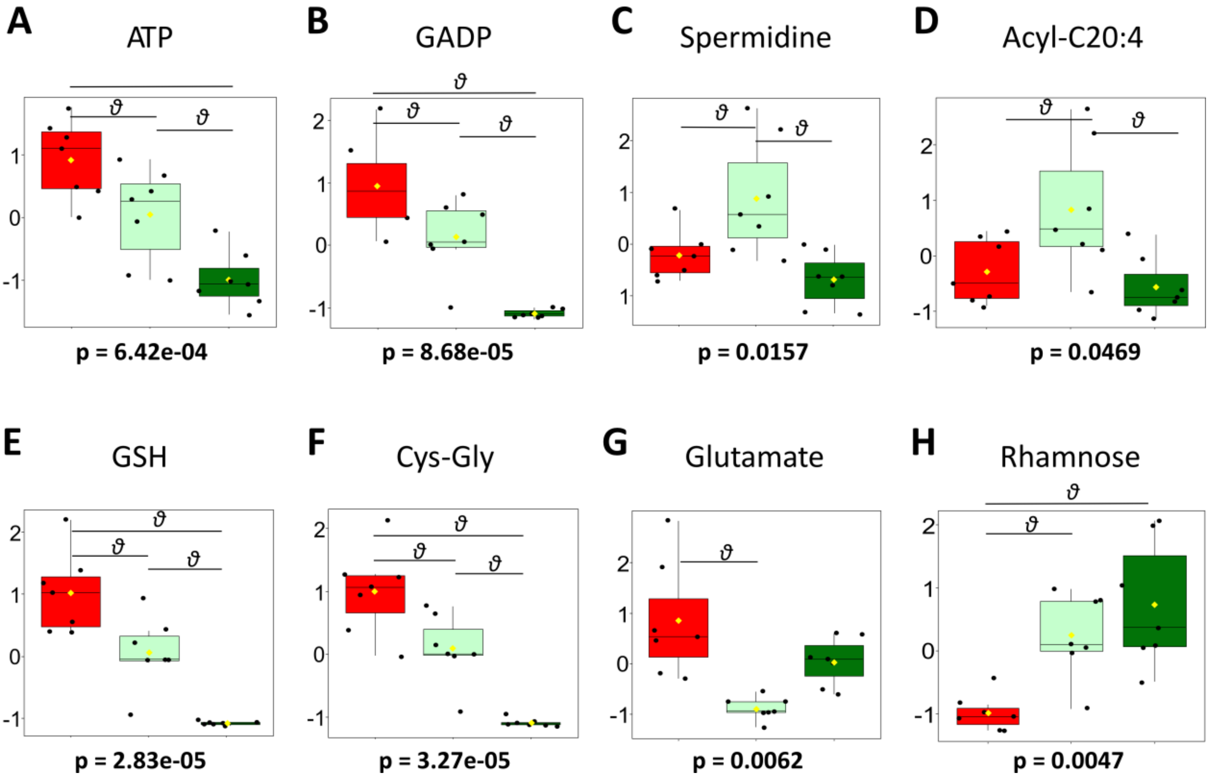

Supplemental Figures

Supplemental Figure 1: Metabolites that significantly vary between flow-sorted short-stored CFSE^{low} and long-stored CFSE^{low} RBCs. Box plots from metabolomic data for metabolites that significantly vary between flow-sorted short stored-CFSE^{low} (Red box plots; n = 7) and long stored-CFSE^{low} (Light green box plots; n = 7) RBCs and for flow-sorted long-stored CFSE^{high} RBCs (Dark green box plots; n = 7). P-values of one-way ANOVA are written under each graph and θ represents a significant difference found by a positive post-hoc test of Tukey's HSD between groups. GADP: glyceraldehyde 3-phosphate; Acyl-C20:4: acylcarnitine with a 20:4 acyl chain; GSH: reduced glutathione; Cys-Gly: cysteinyl-glycine

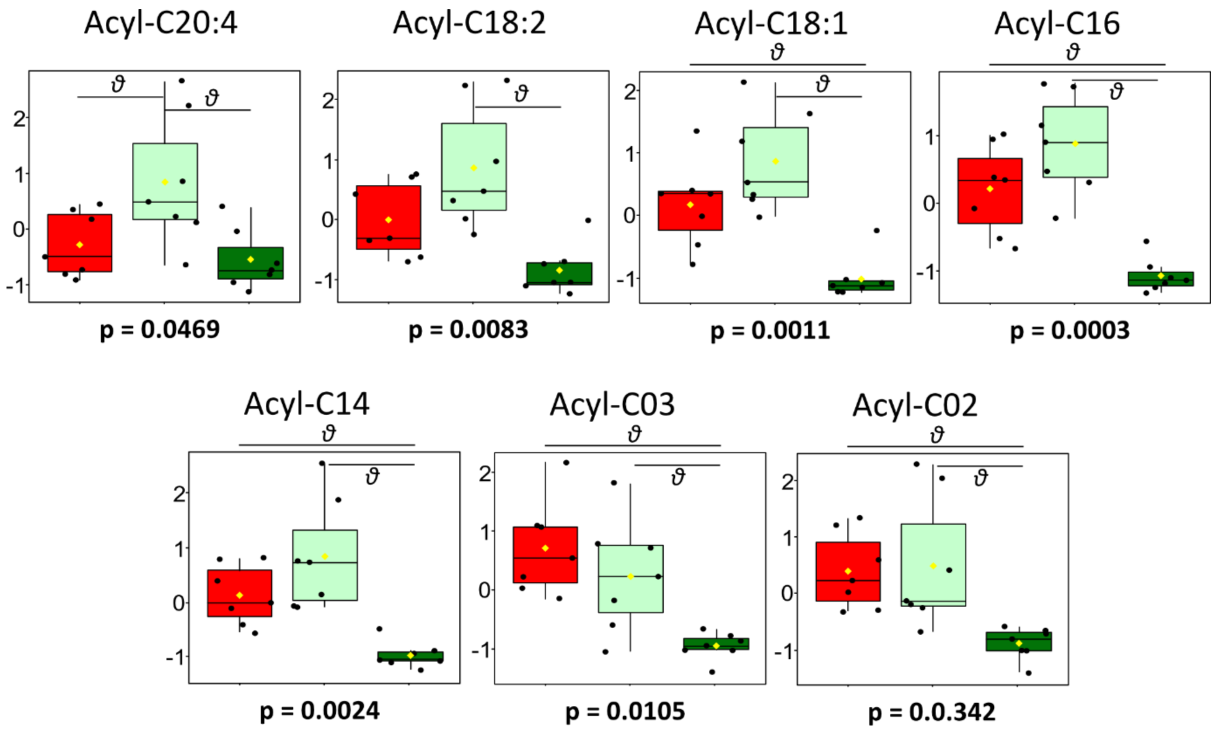

Supplemental Figure 2: Acyl-carnitines that significantly vary between flow-sorted CFSE-stained RBC subsets. Box plots of metabolomic data for metabolites that significantly vary between flow-sorted short stored-CFSE^{low} (Red box plots; n = 7), long stored-CFSE^{low} (Light green box plots; n = 7), and long-stored CFSE^{high} RBCs (Dark green box plots; n = 7). P-values of one-way ANOVA are written under each graph and *θ* represents a significant difference found by a positive post-hoc test of Tukey's HSD between groups. Acyl-CX:Y means acylcarnitine with a X:Y acyl chain

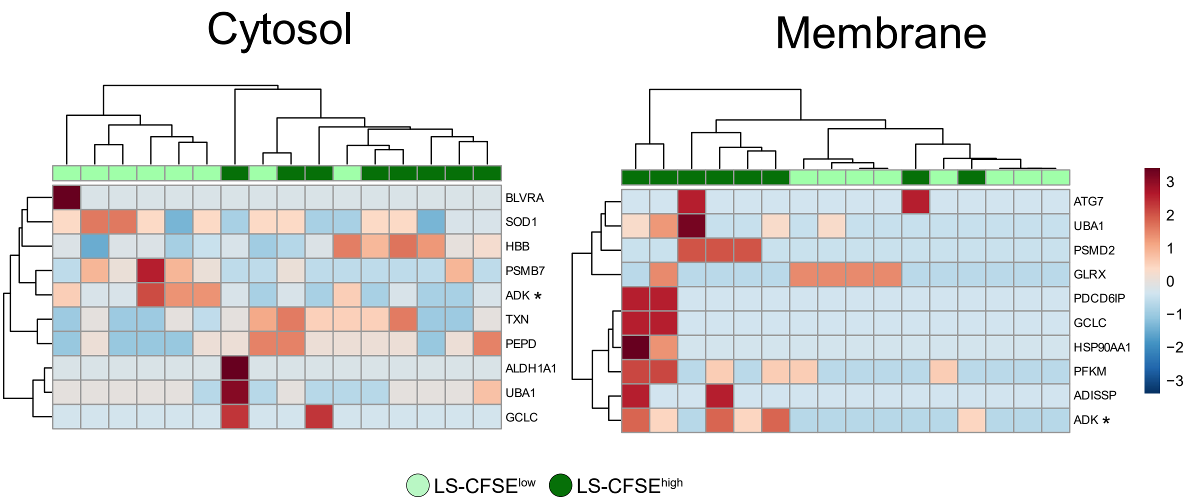

Supplemental Figure 3: Redox proteomics shows increased level of irreversibly oxidized proteins in membrane of the LS-CFSE^{high} subset. Hierarchical clustering analysis of the top 10 irreversibly oxidized proteins ("Cys to DHA") for cytosol (left) and membrane (right) fractions of flow-sorted long-stored CFSE^{low} (light green, n = 8), and long-stored CFSE^{high} RBCs (dark green, n = 8). Significant irreversible oxidation was detected for 1 protein (ADK) in the cytosol and membrane of LS-CFSE^{high} RBCs (* $P < 0.05$ by multiple two-tailed paired t-test).

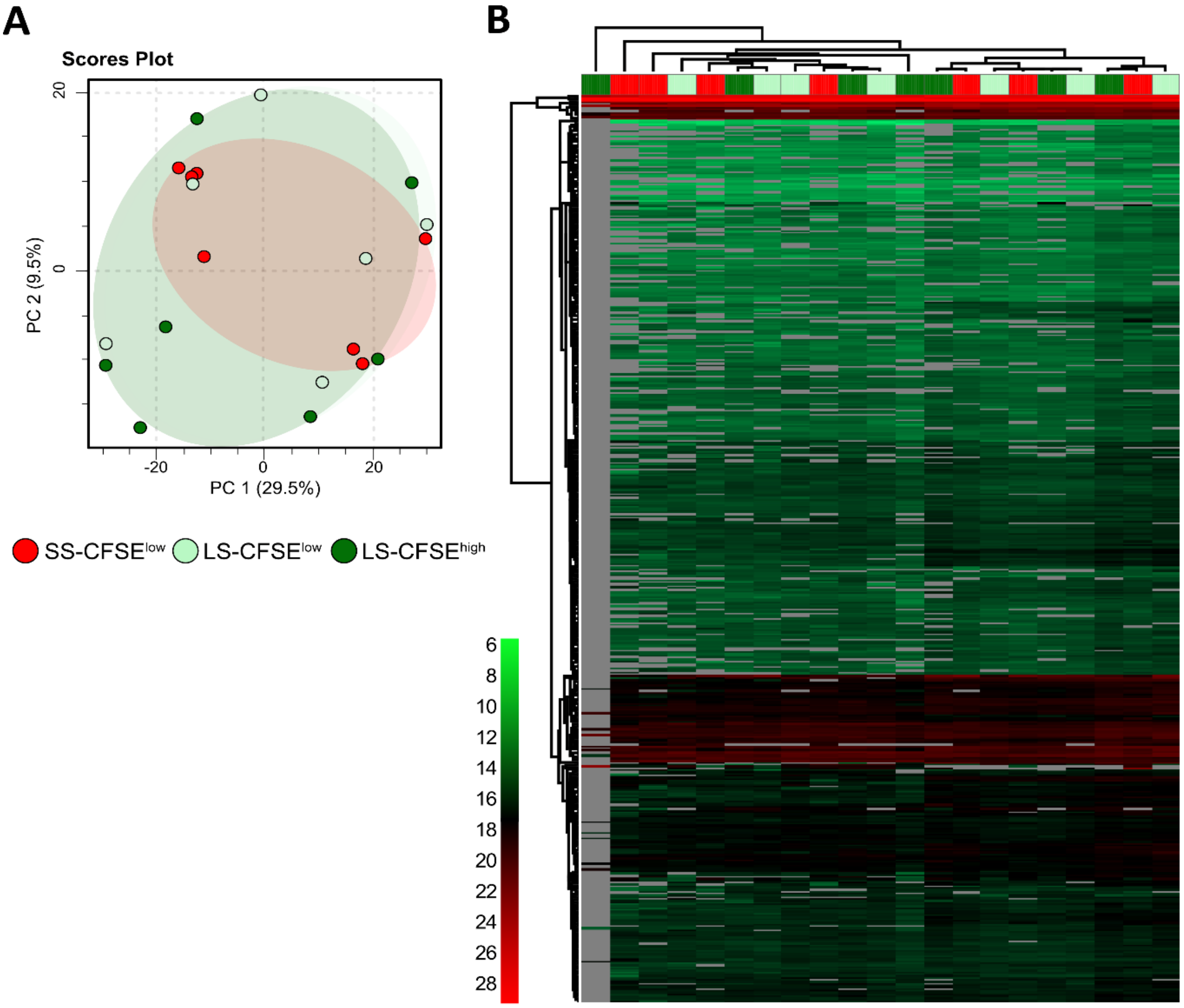

Supplemental Figure 4: Proteomics of intact RBCs reveal no major differences between the 3 CFSE-stained, flow-sorted, RBC subsets analyzed. (A) Principal component analysis of proteomics data for flow-sorted short-stored CFSE^{low} (red, n = 6), long-stored CFSE^{low} (light green, n = 6), and long-stored CFSE^{high} RBCs (dark green, n = 6). **(B)** Hierarchical clustering analysis of the proteins (quantified in at least 70% of the samples in one condition) shows no clustering of the three subsets (data are represented by copy number per cell).

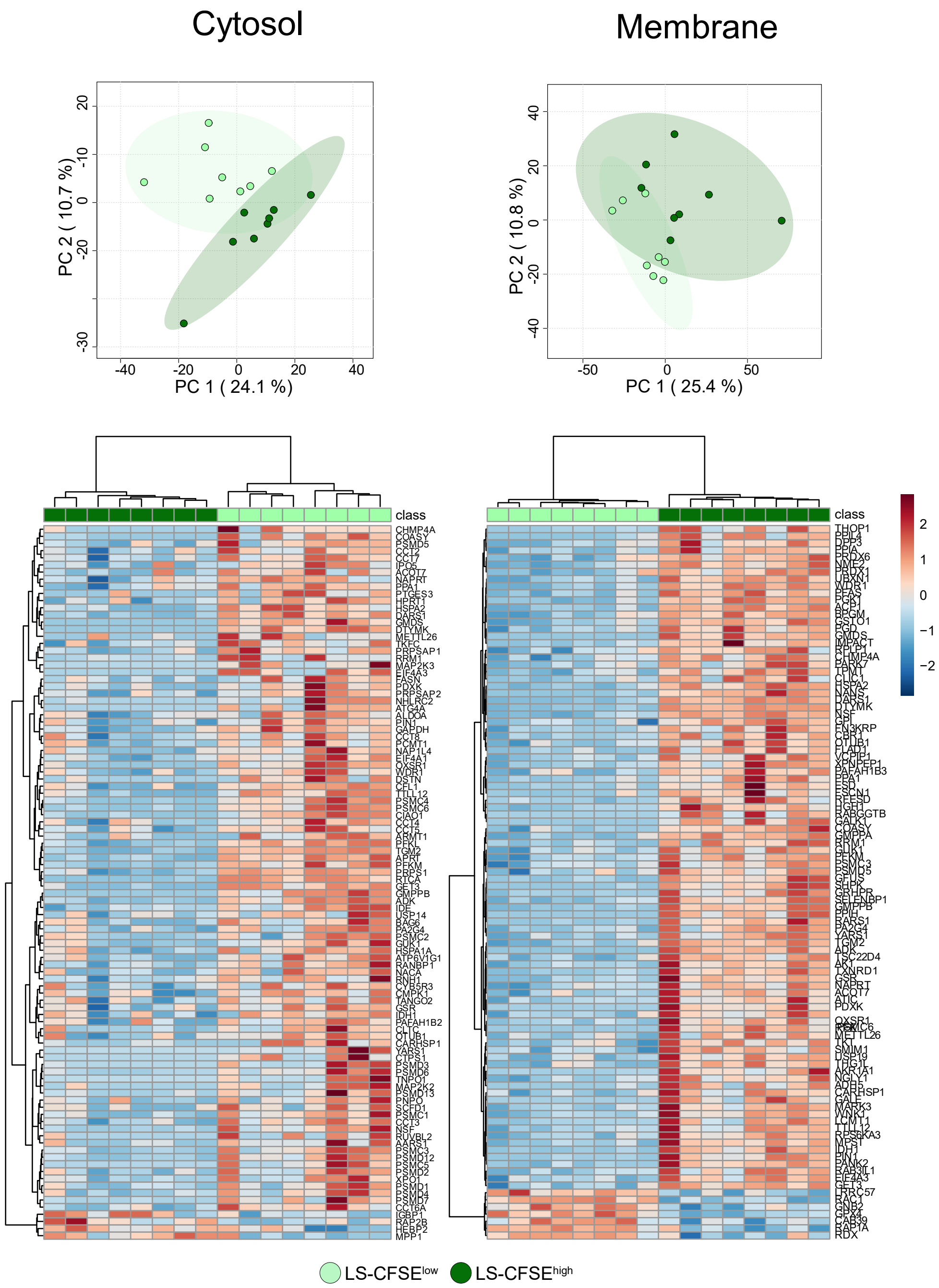

Supplemental Figure 5: Proteomics on RBC cytosol and membrane fractions confirm subset-specific membrane relocation of proteins in long-stored CFSE^{high} RBCs. (Upper panels) Principal component analysis of proteomics data obtained from cytosol (left) and membrane (right) fractions of flow-sorted long-stored CFSE^{low} (light green, n = 8), and long-stored CFSE^{high} RBCs (dark green, n = 8). **(Lower panels)** Hierarchical clustering analysis of the top 100 proteins that vary significantly when comparing the long-stored RBC subsets (by multiple two-tailed paired t-test).

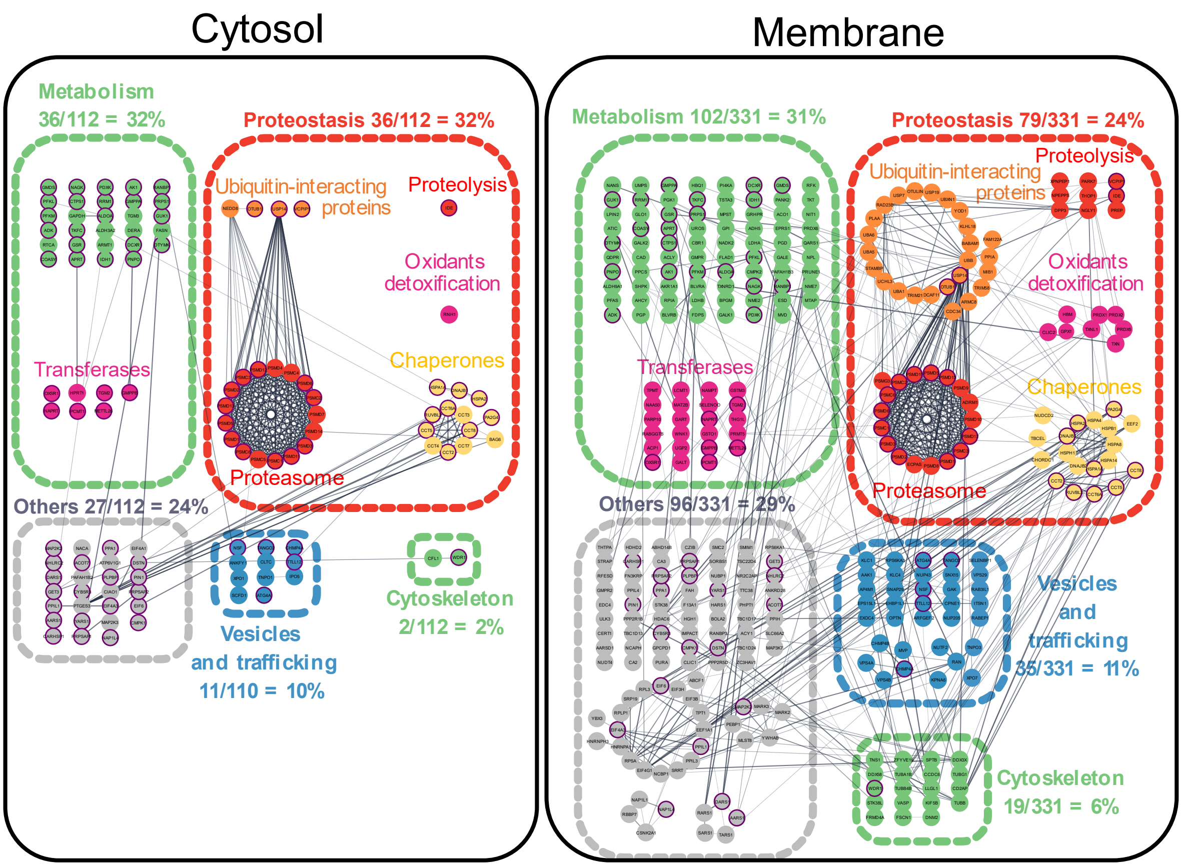

Supplemental Figure 6: Proteomics on membrane and cytosol fractions confirm subset-specific membrane relocation of proteins in long-stored CFSE^{high} RBCs, notably affecting proteins in the "metabolism", "proteostasis" and "vesicles and trafficking" families. Interaction network analysis for proteins significantly decreased in the cytosol fraction **(left panel)** and increased in the membrane fraction **(right panel)** of long-stored CFSE^{high} RBCs (*vs* long-stored CFSE^{low} RBCs). This network was realized using Cytoscape StringApp 3.9. Grey lines show physical interactions between proteins. Purple contours highlight proteins that were identified in both analyses. Colored dotted lines represent the main protein families identified (relative proportion within the significant proteins by multiple t-test) and colored circles represent functional groups within a family.

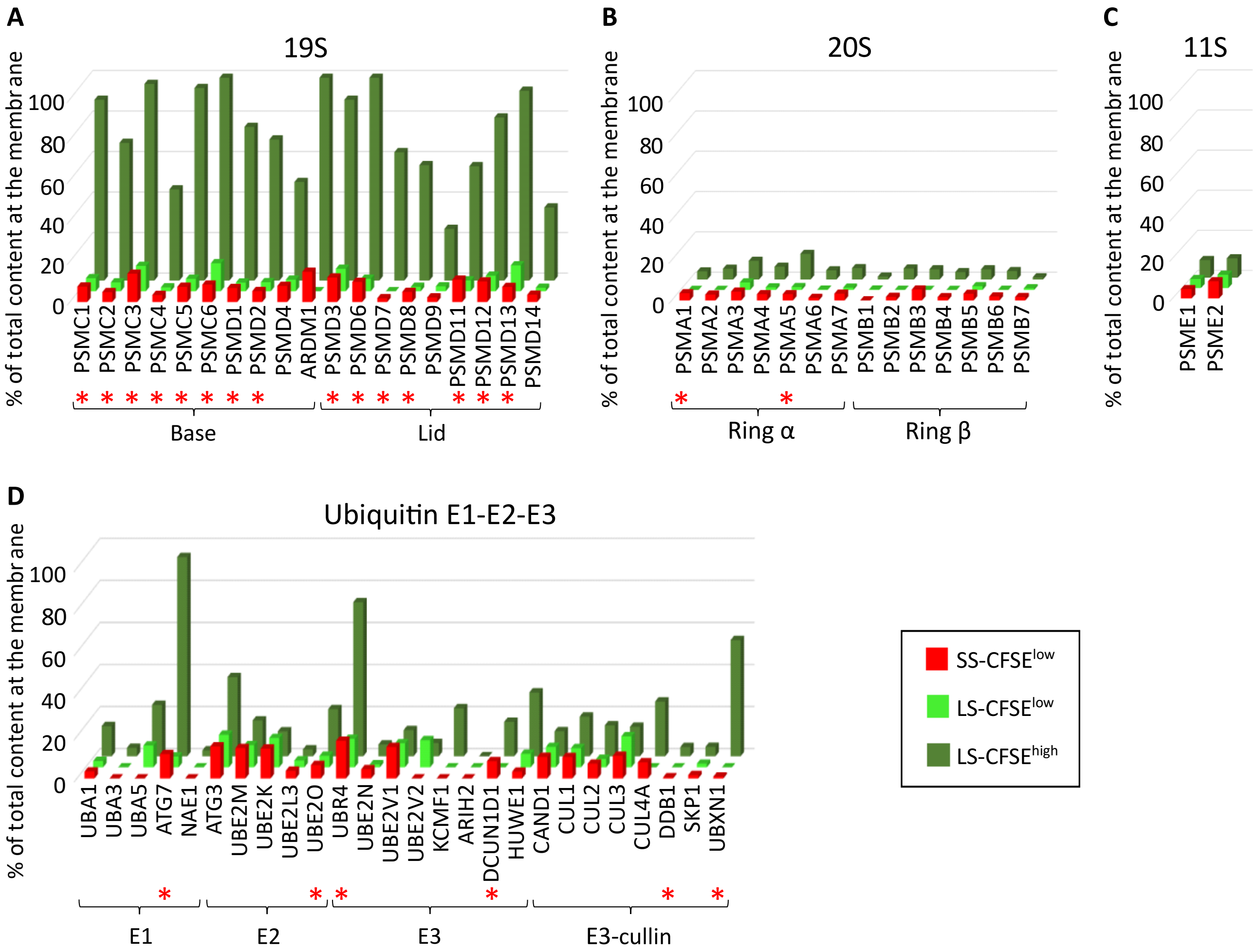

Supplemental Figure 7: Membrane relocation of RBC proteasome and ubiquitin proteins. The proportions of total cellular content found at the membrane of flow-sorted short-stored CFSE^{low} (SS-CFSE^{low}, in red), long-stored CFSE^{low} (LS-CFSE^{low}, in light green), and long-stored CFSE^{high} (LS-CFSE^{high}, in dark green) RBCs for all detected proteins in the 19S **(A)**, 20S **(B)**, and 11S **(C)** proteasome subunits, and the E1, E2, and E3 ubiquitin enzymes **(D)**. Red stars identify proteins that show statistically significant membrane relocation in long-stored CFSE^{high} RBCs (*vs* long-stored CFSE^{low} RBCs).

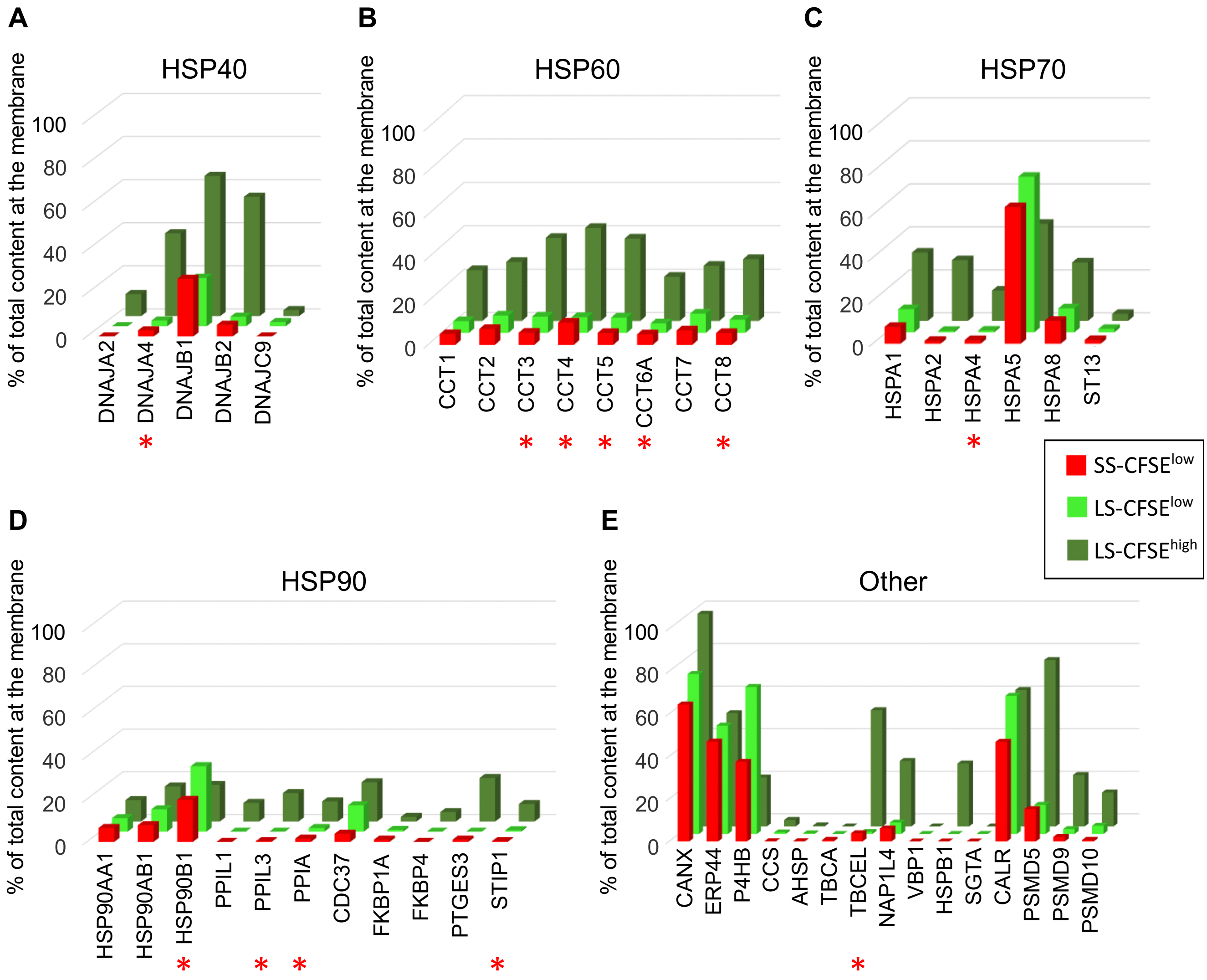

Supplemental Figure 8: Membrane relocation of RBC chaperone proteins. The proportions of the cellular total content found at the membrane of flow-sorted short-stored CFSE^{low} (SS-CFSE^{low}, in red), long-stored CFSE^{low} (LS-CFSE^{low}, in light green), and long-stored CFSE^{high} (LS-CFSE^{high}, in dark green) RBCs for all detected chaperone proteins in the **(A)** HSP40, **(B)** HSP60, (**C**) HSP70, and (**D**) HSP90 families, and (**E**) other chaperone proteins. Red stars identify proteins that show statistically significant membrane relocation in long-stored CFSE^{high} RBCs (*vs* long-stored CFSE^{low} RBCs).

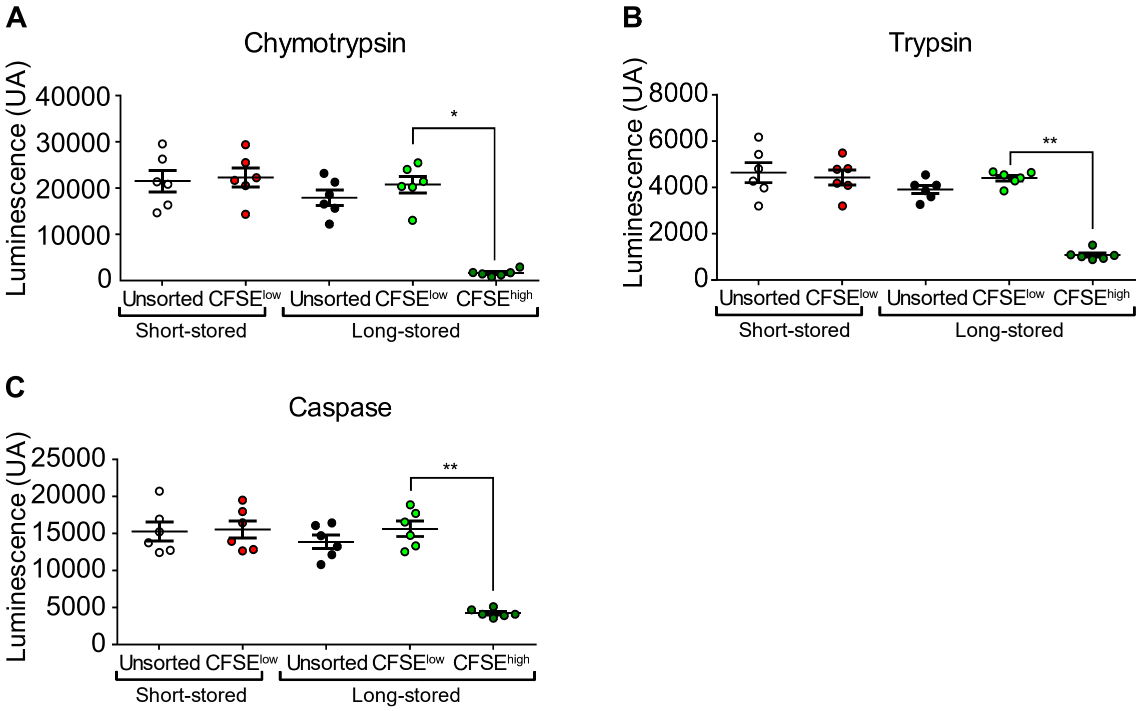

Supplemental Figure 9: Short-stored CFSE^{low} and long-stored CFSE^{low} RBCs have similar levels of proteasomal activity. Chymotrypsin-like (**A**), Trypsin-like (**B**), and Caspase-like (**C**) proteasome activities were measured for CFSE-stained short-stored (unsorted and CFSE^{low}) and long-stored (unsorted, CFSE^{low}, CFSE^{high}) RBC subsets. Data are presented as 6 individual experiments with the mean shown ± SEM. * P <0.05, ** P <0.01 by Friedman one-way ANOVA followed by Dunn's multiple comparison test.

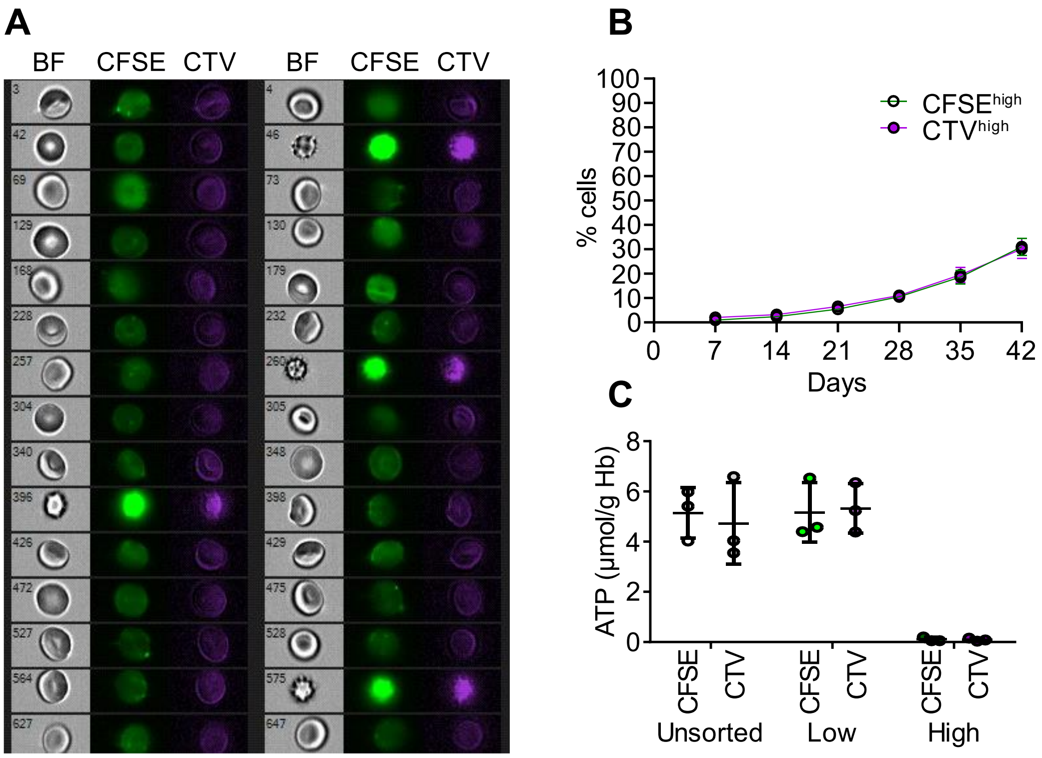

Supplemental Figure 10: The CTV probe shows staining properties similar to CFSE, allowing quantification of morphologically-altered RBCs by flow cytometry in a different fluorescence channel. (A) Representative images of CFSE and CTV co-stained RBCs obtained by ImageStream; BF = Brightfield. **(B)** Quantification of CFSE^{high} and CTV^{high} RBCs cells upon storage of RBC concentrates in SAGM solution for 42 days (n = 8, mean ± SEM). **(C)** Intracellular ATP levels normalized for hemoglobin content in sorted RBC subsets identified by CFSE or CTV staining. Data are presented as 3 individual experiments with the mean shown ± SD. In panels B and C, a two-way ANOVA followed by a Sidak's multiple comparison was performed to compare both staining at each time points or for each subset, respectively; no statistically significant differences were observed.

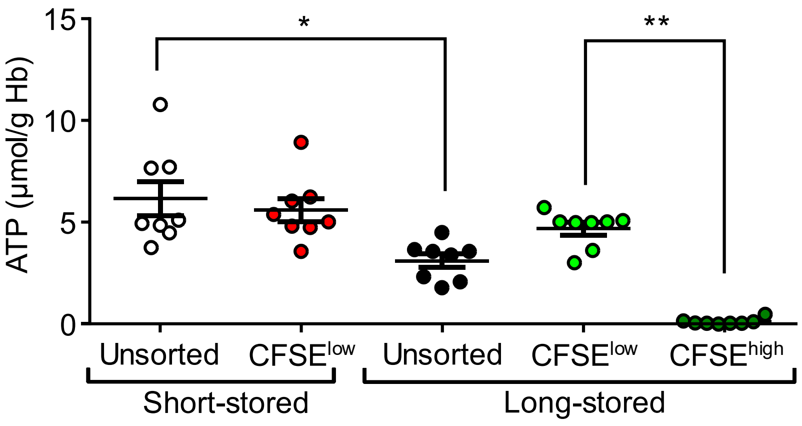

Supplemental Figure 11: Flow-sorted short-stored CFSE^{low} RBCs and long-stored CFSE^{low} RBCs have similar intracellular ATP levels. Intracellular ATP levels normalized for hemoglobin content were evaluated for CFSE-stained short-stored (unsorted and CFSE$^{low)}$ and long-stored (unsorted, CFSE^{low}, CFSE^{high}) RBC subsets. Data are represented as 8 individual experiments with the mean shown ± SEM. * P <0.05, ** P <0.01 by Friedman one-way ANOVA followed by Dunn’s multiple comparison test.

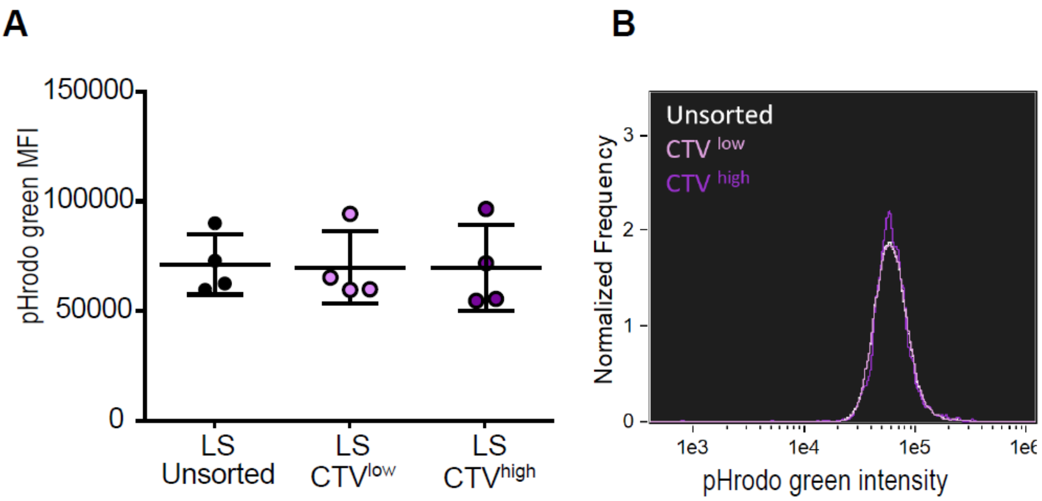

Supplemental Figure 12: Intracellular pH is similar in all long-stored RBCs. Intracellular pH (measured using the pHrodo green probe) was evaluated by imaging flow cytometry for CTV-stained long-stored (unsorted, CTV^{low}, CTV^{high}) RBC subsets. (A) Mean Fluorescence Intensity (MFI); (B) Representative normalized frequency plot for each RBC subset. There was no statistical difference between CTV^{low} and CTV^{high} subsets by Friedman one-way ANOVA followed by Dunn's multiple comparison test.

References

1. Roussel C, et al. Spherocytic shift of red blood cells during storage provides a quantitative whole cell-based marker of the storage lesion. *Transfusion*. 2017;57(4):1007–1018.

2. Perez-Riverol Y, et al. The PRIDE database resources in 2022: a hub for mass spectrometry-based proteomics evidences. *Nucleic Acids Res*. 2022;50(D1):D543–D552.

3. Wiśniewski JR, et al. Universal sample preparation method for proteome analysis. *Nat Methods*. 2009;6(5):359–362.

4. Cox J, et al. Accurate proteome-wide label-free quantification by delayed normalization and maximal peptide ratio extraction, termed MaxLFQ. *Mol Cell Proteomics*. 2014;13(9):2513–2526.

5. Gautier E-F, et al. Absolute proteome quantification of highly purified populations of circulating reticulocytes and mature erythrocytes. *Blood Adv*. 2018;2(20):2646–2657.

6. Tyanova S, et al. The Perseus computational platform for comprehensive analysis of (prote)omics data. *Nat Methods*. 2016;13(9):731–740.

7. Nemkov T, Hansen KC, D'Alessandro A. A three-minute method for high-throughput quantitative metabolomics and quantitative tracing experiments of central carbon and nitrogen pathways. *Rapid Commun Mass Spectrom*. 2017;31(8):663–673.

8. Nemkov T, et al. High-Throughput Metabolomics: Isocratic and Gradient Mass Spectrometry-Based Methods. *Methods Mol Biol*. 2019;1978:13–26.

9. Reisz JA, et al. Untargeted and Semi-targeted Lipid Analysis of Biological Samples Using Mass Spectrometry-Based Metabolomics. *Methods Mol Biol*. 2019;1978:121–135.

10. Issaian A, et al. The interactome of the N-terminus of band 3 regulates red blood cell metabolism and storage quality. *Haematologica*. 2021;106(11):2971–2985.

11. Pang Z, et al. MetaboAnalyst 5.0: narrowing the gap between raw spectra and functional insights. *Nucleic Acids Res*. 2021;49(W1):W388–W396.
